# Supplementary material for: The association between dementia severity and hospitalisation profile in a newly assessed clinical cohort: the South London and Maudsley case register
Source: BMJ Open. 2020 Apr 12;10(4):e035779. doi: 10.1136/bmjopen-2019-035779 (PMC7200045; doi:10.1136/bmjopen-2019-035779)
Supplement: Supplementary data [file bmjopen-2019-035779supp001.pdf]

Table S1: **Twenty most common primary discharge diagnoses amongst individuals with dementia receiving inpatient care in the 12 months after dementia diagnosis (repeat admissions excluded)**

**Primary discharge diagnosis (ICD code)***	**Number of episodes**	**% of total inpatient care episodes**
Other disorders of urinary system (N39)	399	15.4
Pneumonia, organism unspecified (J18)	248	9.6
Fracture of femur (S72)	150	5.8
Senility (R54)	128	4.9
Syncope and collapse (R55)	117	4.5
Other cataract (H26)	107	4.1
Unspecified acute lower respiratory infection (J22)	97	3.7
Cerebral infarction (I63)	86	3.3
Other symptoms & signs involving cognitive function and awareness (R41)	86	3.3
Pain in throat and chest (R07)	78	3.0
Open wound of head (S01)	75	2.9
Unspecified dementia (F03)	73	2.8
Other chronic obstructive pulmonary disease (J44)	71	2.7
Superficial injury of head (S00)	67	2.6
Acute renal failure (N17)	57	2.2
Alzheimer's disease (G30)	56	2.2
Heart failure (I50)	52	2.0
Pneumonitis due to solids and liquids (J69)	49	1.9
Other joint disorders, not elsewhere classified (M25)	46	1.8
Other functional intestinal disorders (K59)	43	1.7

*Three digit ICD-10 discharge diagnoses; repeat admissions for the same diagnosis removed.
Percentages based on 2,596 individuals with at least 1 inpatient admission in the 12 month period after the dementia diagnosis date

Table S2: **ICD chapter level primary discharge diagnoses amongst individuals with dementia receiving inpatient care in the 12 months after dementia diagnosis (repeat episodes excluded)***

**Chapter**		**Number of episodes**	**% of total inpatient care episodes**
**A**	Infectious and parasitic diseases	59	2.3
**B**	Infectious and parasitic diseases	11	0.4
**C**	Cancers	123	4.7
**D**	Benign neoplasms or diseases of the blood	119	4.6
**E**	Endocrine, nutritional and metabolic diseases	122	4.7
**F**	Mental and behavioural disorders	180	6.9
**G**	Diseases of the nervous system	159	6.1
**H**	Diseases of the eye	166	6.4
**I**	Diseases of the circulatory system	542	20.9
**J**	Diseases of the respiratory system	514	19.8
**K**	Diseases of the digestive system	395	15.2
**L**	Diseases of the skin	89	3.4
**M**	Diseases of the musculoskeletal system	214	8.2
**N**	Diseases of the genitourinary system	554	21.3
**R**	Symptoms and signs not elsewhere classified	803	30.9
**S**	Injury, poisoning and certain other external causes	545	21.0
**T**	Injury, poisoning and certain other external causes	100	3.9
**Z**	External causes	140	5.4

*Percentages based on 2,596 individuals with at least 1 inpatient admission in the 12 month period after the dementia diagnosis date. Repeat episodes for the same 3-digit ICD code in the 12-month time period excluded

Table S3: Standardised admissions ratios (95% CI) for hospitalisations in the 12 months following a first dementia diagnosis – by 3 digit ICD-10 code for the primary discharge diagnosis* (repeat admissions excluded)

Primary discharge diagnosis	By dementia severity at first diagnosis					
	All	Mild	Moderate	Severe	Coefficient†	P-value†
**Other disorders of urinary system (N39)**	2.5 (2.3-2.5)	2.3 (2.0-2.7)	2.6 (2.3-3.0)	2.9 (2.2-3.8)	0.29	**0.01**
**Pneumonia, organism unspecified (J18)**	2.0 (1.7-2.3)	1.5 (1.2-1.9)	2.1 (1.8-2.5)	3.0 (2.2-4.1)	0.77	0.07
**Fracture of femur (S72)**	2.4 (2.0-2.8)	2.4 (1.8-3.2)	2.4 (1.9-3.0)	2.5 (1.4-3.9)	0.01	0.8
**Senility (R54)**	2.8 (2.4-3.4)	2.8 (2.0-3.7)	2.8 (2.1-3.5)	3.4 (2.0-5.5)	0.32	0.33
**Syncope and collapse (R55)**	2.8 (2.3-3.4)	2.8 (2.1-3.8)	2.8 (2.1-3.6)	3.0 (1.6-5.1)	0.07	0.44
**Other cataract (H26)**	0.6 (0.5-0.7)	0.5 (0.3-0.7)	0.7 (0.5-0.9)	0.5 (0.3-1.0)	0.04	0.8
**Unspecified acute lower respiratory infection (J22)**	1.9 (1.5-2.3)	1.5 (1.0-2.1)	1.9 (1.4-2.5)	2.8 (1.6-4.6)	0.66	0.12
**Cerebral infarction (I63)**	1.7 (1.3-2.1)	1.4 (0.9-2.1)	1.6 (1.2-2.2)	2.7 (1.5-4.5)	0.65	0.24
**Other symptoms & signs involving cognitive function and awareness (R41)**	5.1 (4.0-6.2)	4.2 (2.7-6.1)	5.7 (4.3-7.6)	4.9 (2.3-9.4)	0.39	0.67
**Pain in throat and chest (R07)**	1.4 (1.1-1.7)	1.7 (1.2-2.3)	1.2 (0.9-1.7)	0.8 (0.3-2.0)	-0.42	**0.03**
**Open wound of head (S01)**	3.0 (2.4-3.7)	3.3 (2.2-4.7)	2.7 (1.9-3.8)	3.2 (1.5-6.1)	-0.04	0.91
**Unspecified dementia (F03)**	8.6 (6.7-10.8)	5.4 (3.1-8.6)	10.7 (7.9-14.2)	9.6 (4.4-18.3)	2.11	0.46
**Other chronic obstructive pulmonary disease (J44)**	1.1 (0.8-1.4)	1.1 (0.7-1.6)	1.1 (0.8-1.5)	0.7 (0.2-1.7)	-0.18	0.35
**Superficial injury of head (S00)**	3.4 (2.6-4.3)	3.3 (2.1-5.0)	3.1 (2.1-4.4)	4.9 (2.5-8.8)	0.79	0.41
**Acute renal failure (N17)**	2.2 (1.7-2.9)	1.6 (0.9-2.6)	2.4 (1.6-3.3)	3.7 (1.8-6.8)	1.05	0.1
**Alzheimer's disease (G30)**	10.4 (7.9-13.6)	7.5 (4.2-12.3)	10.5 (7.0-15.0)	20.5 (10.6-35.8)	6.5	0.19
**Heart failure (I50)**	0.9 (0.6-1.1)	0.6 (0.4-1.1)	1.1 (0.7-1.5)	0.6 (0.2-1.6)	-0.02	0.96
**Pneumonitis due to solids and liquids (J69)**	3.0 (2.2-4.0)	1.6 (0.8-3.0)	3.7 (2.5-5.2)	4.6 (2.0-9.0)	1.46	0.14
**Other joint disorders, not elsewhere classified (M25)**	1.7 (1.3-2.3)	1.3 (0.7-2.2)	1.8 (1.2-2.7)	2.8 (1.2-5.6)	0.79	0.09
**Other functional intestinal disorders (K59)**	1.5 (1.1-2.0)	1.8 (1.1-2.8)	1.4 (0.9-2.2)	0.7 (0.1-2.4)	-0.58	0.12

*Based on 2,617 individuals with at least one hospitalisation in the pre index period and 2,596 individuals in the post index period

†The regression coefficient indicates the increase or decrease in SAR associated with a one-step increase in severity of dementia. See Statistical analyses section for the linear model relating these variables.

Table S4: **Standardised admissions ratios (95% CI) by ICD chapters (repeat admissions excluded)***

	Chapter	All	Mild	Moderate	Severe	Coefficient†	p-value †
**A**	Infectious and parasitic diseases	1.6 (1.2-2.0)	1.0 (0.6-1.7)	1.8 (1.3-2.5)	2.2 (1.0-4.2)	0.58	0.11
**B**	Infectious and parasitic diseases	1.7 (0.9-3.1)	0.8 (0.1-3.0)	2.2 (0.9-4.5)	2.9 (0.4-10.6)	1.06	0.11
**C**	Cancers	0.6 (0.5-0.7)	0.7 (0.5-0.8)	0.6 (0.4-0.7)	0.3 (0.1-0.7)	-0.17	0.19
**D**	Benign neoplasms or diseases of the blood	1.0 (0.8-1.2)	1.1 (0.9-1.5)	1.0 (0.8-1.3)	0.5 (0.2-1.1)	-0.32	0.22
**E**	Endocrine, nutritional and metabolic diseases	2.0 (1.7-2.4)	2.0 (1.5-2.7)	1.9 (1.5-2.5)	2.5 (1.4-4.1)	0.27	0.39
**F**	Mental and behavioural disorders	5.8 (4.9-6.7)	5.0 (3.8-6.4)	6.2 (5.0-7.5)	6.6 (4.1-9.9)	0.8	0.19
**G**	Diseases of the nervous system	2.5 (2.1-2.9)	2.3 (1.7-2.9)	2.6 (2.1-3.3)	3.1 (1.9-4.7)	0.4	0.03
**H**	Diseases of the eye	0.6 (0.6-0.8)	0.6 (0.5-0.8)	0.7 (0.6-0.9)	0.5 (0.2-0.8)	-0.07	0.65
**I**	Diseases of the circulatory system	1.2 (1.1-1.2)	1.3 (1.1-1.5)	1.2 (1.1-1.4)	1.1 (0.8-1.4)	-0.11	0.08
**J**	Diseases of the respiratory system	1.7 (1.5-1.7)	1.3 (1.1-1.6)	1.8 (1.6-2.0)	2.2 (1.8-2.8)	0.45	0.03
**K**	Diseases of the digestive system	1.0 (0.9-1.0)	1.1 (1.0-1.3)	0.9 (0.8-1.1)	0.7 (0.4-1.0)	-0.25	0.06
**L**	Diseases of the skin	1.1 (0.9-1.4)	1.1 (0.8-1.5)	1.0 (0.7-1.4)	1.8 (1.0-2.9)	0.34	0.42
**M**	Diseases of the musculoskeletal system	0.9 (0.8-1.1)	1.0 (0.8-1.3)	0.9 (0.7-1.1)	1.0 (0.7-1.5)	-0.01	0.95
**N**	Diseases of the genitourinary system	1.8 (1.7-1.8)	1.6 (1.4-1.9)	1.9 (1.7-2.1)	2.2 (1.7-2.8)	0.28	0.01
**R**	Symptoms and sign not elsewhere classified	1.9 (1.7-1.9)	1.9 (1.7-2.1)	1.9 (1.7-1.9)	1.7 (1.3-2.1)	-0.12	0.29
**S**	Injury, poisoning and certain other external causes	2.3 (2.1-2.3)	2.3 (2.0-2.7)	2.2 (1.9-2.4)	2.4 (1.8-3.1)	0.03	0.86
**T**	Injury, poisoning and certain other external causes	1.5 (1.3-1.9)	1.5 (1.1-2.1)	1.8 (1.3-2.3)	0.7 (0.2-1.7)	-0.37	0.49
**Z**	External causes	1.1 (0.9-1.3)	1.5 (1.2-1.9)	0.9 (0.7-1.2)	0.8 (0.4-1.4)	-0.34	0.22
Total		1.4 (1.4-1.4)	1.4 (1.3-1.4)	1.4 (1.4-1.4)	1.4 (1.3-1.4)	0.01	0.49

*Based on 2,596 individuals in the post index period †The regression coefficient indicates the increase or decrease in SAR associated with a one-step increase in severity of dementia. See Statistical analyses section for the linear model relating these variables.

Gungabissoon U, *et al. BMJ Open* 2020; 10:e035779. doi: 10.1136/bmjopen-2019-035779
