# Supplementary material for: The association between dementia severity and hospitalisation profile in a newly assessed clinical cohort: the South London and Maudsley case register
Source: BMJ Open. 2020 Apr 12;10(4):e035779. doi: 10.1136/bmjopen-2019-035779 (PMC7200045; doi:10.1136/bmjopen-2019-035779)
Supplement: Author's manuscript [file bmjopen-2019-035779.draft_revisions.pdf]

BMJ Open

BMJ Open is committed to open peer review. As part of this commitment we make the peer review history of every article we publish publicly available.

When an article is published we post the peer reviewers' comments and the authors' responses online. We also post the versions of the paper that were used during peer review. These are the versions that the peer review comments apply to.

The versions of the paper that follow are the versions that were submitted during the peer review process. They are not the versions of record or the final published versions. They should not be cited or distributed as the published version of this manuscript.

BMJ Open is an open access journal and the full, final, typeset and author-corrected version of record of the manuscript is available on our site with no access controls, subscription charges or pay-per-view fees (http://bmjopen.bmj.com).

If you have any questions on BMJ Open's open peer review process please email
info.bmjopen@bmj.com

BMJ Open

The relationship between dementia severity and hospitalisation profile in a newly assessed clinical cohort: The South London and Maudsley Case Register

Journal:	*BMJ Open*
Manuscript ID	bmjopen-2019-035779
Article Type:	Original research
Date Submitted by the Author:	15-Nov-2019
Complete List of Authors:	Gungabissoon, Usha; GSK, Worldwide Epidemiology<br>Perera, Gayan; Institute of Psychiatry, Psychological Medicine<br>Galwey, Nicholas; Target Sciences, GSK R&D<br>Stewart, Robert; South London and Maudsley NHS Foundation Trust
Keywords:	Dementia < NEUROLOGY, EPIDEMIOLOGY, HEALTH ECONOMICS

SCHOLARONE™
Manuscripts

I, the Submitting Author has the right to grant and does grant on behalf of all authors of the Work (as defined in the below author licence), an exclusive licence and/or a non-exclusive licence for contributions from authors who are: i) UK Crown employees; ii) where BMJ has agreed a CC-BY licence shall apply, and/or iii) in accordance with the terms applicable for US Federal Government officers or employees acting as part of their official duties; on a worldwide, perpetual, irrevocable, royalty-free basis to BMJ Publishing Group Ltd ("BMJ") its licensees and where the relevant Journal is co-owned by BMJ to the co-owners of the Journal, to publish the Work in this journal and any other BMJ products and to exploit all rights, as set out in our licence.

The Submitting Author accepts and understands that any supply made under these terms is made by BMJ to the Submitting Author unless you are acting as an employee on behalf of your employer or a postgraduate student of an affiliated institution which is paying any applicable article publishing charge ("APC") for Open Access articles. Where the Submitting Author wishes to make the Work available on an Open Access basis (and intends to pay the relevant APC), the terms of reuse of such Open Access shall be governed by a Creative Commons licence – details of these licences and which Creative Commons licence will apply to this Work are set out in our licence referred to above.

Other than as permitted in any relevant BMJ Author's Self Archiving Policies, I confirm this Work has not been accepted for publication elsewhere, is not being considered for publication elsewhere and does not duplicate material already published. I confirm all authors consent to publication of this Work and authorise the granting of this licence.

For peer review only

The relationship between dementia severity and hospitalisation profile in a newly assessed clinical cohort: The South London and Maudsley Case Register

Authors:

Usha Gungabissoon[1,2], M.Sc.; Gayan Perera[1], Ph.D.; Nicholas Galwey[3], Ph.D.; Robert Stewart[1,4], M.D., FRCPsych

[1]Institute of Psychiatry, Psychology and Neuroscience (IOPPN), King's College London, London, United Kingdom (UK)

[2]Epidemiology, Global Medical, GlaxoSmithKline (GSK) R&D, London, UK

[3]Target Sciences, GSK R&D, London, UK

[4]South London and Maudsley NHS Foundation Trust, London, UK

Corresponding author:

Usha Gungabissoon

Department of Psychological Medicine (PO84), Institute of Psychiatry, Psychology and Neuroscience, King's College London. De Crespigny Park, London SE5 8AF

Email: usha.gungabissoon@kcl.ac.uk

Phone: +442089902469

Word count (excluding title page, abstract, references, figures and tables): 2837 words

For peer review only - http://bmjopen.bmj.com/site/about/guidelines.xhtml

Abstract

Background

Hospitalisation is a significant element in the cost of dementia care; however, there is sparse literature describing the most common causes of hospitalisation in people with newly diagnosed dementia, and no previous study has evaluated these by the severity of dementia.

Objective

To evaluate the frequency and causes of hospitalisations in the year following a dementia diagnosis and variation by severity of cognitive impairment at diagnosis.

Design

Retrospective observational cohort study.

Methods

We identified the most common causes of hospitalisation in people with a dementia diagnosis, using data from a large London mental healthcare case register linked to a national hospitalisation database. We also calculated age- and gender-standardised admission ratios by dementia severity (mild/moderate/severe; based on recorded cognitive function at the time of diagnosis) relative to the catchment population within the same geography.

Results

Of the 5,218 patients with dementia, 2,596 (49.8%) patients were hospitalised in the year following diagnosis. The likelihood of an admission, duration of hospitalisation, and mortality rate increased with dementia severity. After excluding re-admissions for the same cause, the

For peer review only

most common hospitalisation discharge diagnoses were urinary system disorders, pneumonia, and fracture of femur. Patients with dementia were hospitalised more than the catchment population for most of the discharge diagnoses evaluated, and standardised admission ratios for urinary and respiratory disorders were higher in those with more severe dementia at diagnosis.

Conclusions

Understanding the factors influencing health service utilisation in dementia is necessary to inform care needs and guide future healthcare resource planning and allocation. This also highlights the need to develop specific strategies for those causes of hospitalisation that are most amenable to prevention in dementia. Our findings are of importance given that the prevalence of dementia is increasing.

Key words: burden, dementia, epidemiology, healthcare utilisation, hospitalisation

For peer review only

For peer review only - http://bmjopen.bmj.com/site/about/guidelines.xhtml

Strengths and limitations of this study

- We evaluated hospitalisation in a large and representative sample of newly diagnosed cases of dementia in a London mental healthcare case register linked to a national hospitalisation database providing near-complete outcome ascertainment.
- Severity of dementia at the time of diagnosis was determined from the Mini Mental State Examination score or Health of the Nation Outcome Scales impairment score, which has acceptable/good psychometric properties and correlates with MMSE measurement.
- We obtained the analysed samples from a single service provider in a single London borough, which may limit the generalisability of our findings to other settings.
- The analyses did not account for comorbidities, use of medications, institutional residence or socioeconomic status, aetiology of dementia or hospitalisation rates prior to the diagnosis of dementia.

For peer review only

For peer review only - http://bmjopen.bmj.com/site/about/guidelines.xhtml

INTRODUCTION

In the United Kingdom (UK) there are around 850,000 people living with dementia and the associated annual healthcare costs are around £4.3 billion; major contributing factors to costs include disease severity and hospitalisation.[1] Compared to age-matched controls, people with dementia are more likely to require an acute hospital admission,[2, 3] more hours of care and longer hospital stays.[4] Hospitalisation also often represents a pivotal event for people with dementia due to increased risk of admission to long-term institutional care, functional decline, mortality, loss of independence, and impact on caregivers.[5] Despite this impact and the potential for preventative intervention, few studies have evaluated the extent to which the risk and causes of hospital admission vary by severity of dementia. We sought to address this in a large cohort, drawn from a mental healthcare case register linked to national hospitalisation episodes.

For peer review only

For peer review only - http://bmjopen.bmj.com/site/about/guidelines.xhtml

METHODS

Study design

This retrospective observational cohort study involved analysis of newly presenting patients with a dementia diagnosis. We evaluated hospital episodes in the 12-month period following a dementia diagnosis.

Study setting and data source

The Clinical Record Interactive Search (CRIS) data resource was used to identify dementia cases. CRIS provides research access to anonymised electronic health records (EHR) information from the South London and Maudsley NHS Foundation Trust (SLaM). SLaM provides mental healthcare, including dementia assessment and management, for a south London catchment containing approximately 1.2 million residents; EHRs were implemented across all SLaM services from 2006.[6] CRIS data are linked to mortality records and national Hospital Episode Statistics (HES) described below. The Oxfordshire Research Ethics Committee C (reference 18/SC/0372) has approved CRIS at the Maudsley as a data resource for secondary analysis.

Routine diagnoses recorded in SLaM are structured according to the WHO International Classification of Diseases 10th edition (ICD-10) and are supplemented in CRIS, by a natural language processing algorithm ascertaining diagnoses recorded in correspondence and other text fields.[6, 7] Hospital outcomes (hospital admissions and its cause) for patients with dementia were obtained from the HES-CRIS linkage. HES contains details of all inpatient admissions at NHS hospitals in England.[8] Discharge diagnoses are recorded as ICD-10 codes and are available for each hospitalisation episode. Additionally, a subset of the HES database

For peer review only - http://bmjopen.bmj.com/site/about/guidelines.xhtml

detailing hospitalisations for all residents within SLaM's catchment was used in this analysis to generate expected rates for standardisation.

Study participants

We aimed to identify people with newly diagnosed dementia and retrieved records from CRIS for patients with a first recorded diagnosis of dementia between 1 January 2008 and 31 December 2012. We restricted the sample to individuals aged ≥65 years at the time of dementia diagnosis, to those with a measure of cognition within six months of their dementia diagnosis, and to those with a Mini Mental State Examination score (MMSE) score of <28 or Health of the Nation Outcome Scales (HoNoS) cognitive impairment score >1. Patients who were active to acute hospital liaison services at the time of initial diagnosis were excluded since they reflected cases whose dementia diagnosis might have been precipitated by a hospitalisation and who would have an accompanying higher risk of further events.

Covariates

Age at diagnosis (in five-year bands), sex, ethnicity, and dementia severity (within six months of dementia diagnosis) were extracted from CRIS. Dementia severity was estimated primarily from the MMSE recorded closest to the dementia diagnosis date. If no MMSE score was present within six months of the diagnosis date, the closest-recorded cognitive impairment subscale of HoNOS was used if data on this were available within the six-month period around dementia diagnosis. HoNOS is a clinician-rated instrument usually completed at first assessment, which contains subscales rated 0 (no problem) to 4 (severe or very severe problem), has acceptable/good psychometric properties and correlates with MMSE measurement.[9,10] Dementia severity was categorised as mild, moderate, or severe based on

For peer review only - http://bmjopen.bmj.com/site/about/guidelines.xhtml

MMSE scores of 21-27, 10-20 and 0-9 which is similar to cut-offs used by NICE, or a HoNOS cognitive impairment subscale score of 2, 3, or 4, respectively.[11]

Acute general hospital inpatient admissions for cases were obtained for the 12-month period following the date of dementia diagnosis. Follow-up was censored at the earliest of 31 March 2013 or date of death. Hospitalisations were defined from HES episodes, combining contiguous episodes (i.e. where start and end dates were on the same day). Length of hospital stay (LOS) was defined as the number of days from admission to discharge. The three-digit primary ICD-10 discharge diagnosis was obtained for cases and the catchment population for each hospitalisation. All diagnoses were also grouped at the highest level (letter) ICD-10 code, in line with ICD-10-chapter classifications, broadly related to the body system affected.

Hospitalisations for the catchment population from January 2008 to March 2012 were extracted, and the age and sex profile of the catchment was obtained from the UK Census data for the same time frame.

Statistical analyses

Microsoft Excel and Stata 13.0 were used for analyses. Following a descriptive analysis, we calculated the cumulative incidence of general hospital admissions in the dementia cohort accounting for person-years (py) of follow-up. Age- and sex-standardised admission ratios (SARs) with 95% confidence intervals (CI) were calculated for each of the 20 most frequent causes of hospital admission for the 12-month period from the dementia diagnosis based on the three-digit ICD-10 codes. The SAR was defined as the ratio of the observed number of admissions in the dementia cohort to the number expected, derived from the same age and sex

For peer review only - http://bmjopen.bmj.com/site/about/guidelines.xhtml

specific rates in the SLaM catchment. SARs were also calculated for diagnoses at ICD-10-chapter level. SARs were then calculated separately for each category of dementia severity at the time of dementia diagnosis. Linear regression models, with SAR as the dependent variable and dementia stage (mild, moderate, or severe) as the exposure variable, were fitted to quantify the relationship between the severity of dementia at diagnosis and SARs for specific causes of admission of interest (three-digit ICD-10 code and by chapter), using the slope of the regression (direction and magnitude) to describe the trend across dementia stages. In a sensitivity analysis, we excluded repeat admissions (defined as repeat admissions for the same three-digit ICD-10 code in the 12-month period of interest).

For peer review only

RESULTS

A total of 5,815 individuals with a new dementia diagnosis were identified, of whom 343 did not meet the inclusion criteria for MMSE or HoNOS score, and 254 did not have an extractable measure of cognition within the observation window. The remaining 5,218 patients with dementia were included in the analysis. For the majority of the patients (N=4,413; 84.6%), the classification of severity of dementia was based on an MMSE score. For the remaining patients (N=805; 15.4%) severity of dementia was classified using a HoNOS cognitive measure.

The mean age (SD) of the included patients was 82.2 (7.0) years, and almost two-thirds of the patients were women (N=3,338; 64.0%); descriptive characteristics of the analysed cohort by the severity of dementia are displayed in Table 1. At the time of dementia diagnosis, 39.4% were classified as mild, 50.1% as moderate, and 10.4% as severe. The 12-month mortality rate from dementia diagnosis was 15.6% overall and 10.9% for mild severity, 17.5% for moderate, and 24.6% for severe. Approximately half of the patients with dementia were hospitalised in this period (Table 1). While the mean number of admissions did not differ substantially between the dementia severity groups, the likelihood of at least one admission and median duration of inpatient stay (for all admissions in the 12-month period) increased with higher severity of dementia at diagnosis. Patients with dementia were hospitalised 30% more than the catchment population (SAR: 1.3, 95% CI, 1.2-1.3) during the 12-month follow-up and this SAR was similar across dementia severities: mild 1.2 (95% CI, 1.2-1.2), moderate 1.3 (95% CI, 1.3-1.3), and severe 1.3 (95% CI, 1.2-1.3).

For peer review only - http://bmjopen.bmj.com/site/about/guidelines.xhtml

Table 1: Descriptive characteristics of analysis population

	Dementia Severity			All
	Mild	Moderate	Severe	
	N=2,057	N=2,616	N=545	N=5,218
**Mean age (SD)**	81.7 (6.7)	82.6 (7.1)	82.4 (7.5)	82.2 (7.0)
**Sex N (%)**				
Male	765 (37.2)	926 (35.4)	189 (34.7)	1,880 (36.0)
Female	1,292 (62.8)	1,690 (64.6)	356 (65.3)	3,338 (64.0)
**Ethnicity N (%)**				
Afro-Caribbean	219 (10.6)	400 (15.3)	87 (16)	706 (13.5)
Asian	87 (4.2)	105 (4.0)	30 (5.5)	222 (4.3)
European	1,683 (81.8)	2,015 (77.0)	394 (72.3)	4,092 (78.4)
Other	68 (3.3)	96 (3.7)	34 (6.2)	198 (3.8)
**Hospitalisation (12 months after diagnosis)**				
Number of patients with ≥1 inpatient admission	986	1,328	282	2,596
Number of patients per 100 py (95% CI)	54.6 (51.3, 58.1)	60.0 (56.9, 63.3)	63.3 (56.4, 71.2)	58.1 (55.9, 60.4)
Mean number of admissions	2.5	2.4	2.4	2.4
Median (IQR) inpatient stay	2 (1, 11)	3 (1, 13)	5 (1, 13)	3 (1, 12)

IQR, inter quartile range; py, person years; SD, standard deviation

The 20 most common three-digit primary discharge diagnoses for hospitalisation episodes in the year following dementia diagnosis are shown in Table 2. The most common post-dementia discharge diagnosis was chronic renal failure, accounting for 27% of the admissions amongst those hospitalised, followed by disorders of the urinary system (21%). When re-admissions for the same cause were excluded, the most common causes of hospitalisation were disorders of

the urinary system, pneumonia, and fracture of the femur, accounting for 15%, 10%, and 6% of admissions, respectively (Table S1). Considering primary discharge diagnoses at ICD-10 chapter level, the highest proportions of admissions were observed in specific diseases of the genitourinary, circulatory and respiratory systems, accounting for 54%, 25%, and 22% of the hospitalisation episodes, respectively (Table 3); however, the broader chapters for 'other symptoms/signs' and 'injury/external cause events' were also common, accounting for 35% and 22% of admissions, respectively. After exclusion of repeat admissions (Table S2), the highest proportions of admissions at a chapter level were in specific diseases of the genitourinary, circulatory and respiratory systems each accounting for around 20% of the hospitalisation episodes. The broader chapters for 'other symptoms/signs' and 'injury/external cause events', accounted for 31% and 21% of admissions, respectively, after exclusion of repeat admissions at a three-digit ICD level.

For peer review only

For peer review only - http://bmjopen.bmj.com/site/about/guidelines.xhtml

Table 2: Standardised admissions ratios by discharge diagnosis (20 most common ICD-10 3-digit code) for hospitalisations in the 12 month period following a dementia diagnosis

3-digit ICD primary discharge diagnosis	Number of hospitalisations with this discharge diagnosis	Proportion (%) of episodes with this discharge diagnosis	Standardised admission ratio (95% CI) by dementia severity at diagnosis				Regression coefficient*	P-value*
			All cases	Mild N=986	Moderate N=1,328	Severe N=282		
**Chronic renal failure (N18)**	710	27.3	1.0 (0.9-1.0)	0.8 (0.7-0.9)	1.0 (0.9-1.1)	2.2 (1.9-2.6)	0.71	0.26
**Other disorders of urinary system (N39)**	532	20.5	2.7 (2.5-2.7)	2.4 (2.1-2.8)	2.9 (2.6-3.2)	4.8 (3.9-5.8)	1.18	0.21
**Pneumonia, organism unspecified (J18)**	263	10.1	1.9 (1.7-2.2)	1.4 (1.1-1.8)	2.1 (1.8-2.5)	4.5 (3.5-5.7)	1.56	0.2
**Fracture of femur (S72)**	156	6.0	2.4 (2.0-2.8)	2.4 (1.8-3.1)	2.3 (1.9-2.9)	3.1 (1.9-4.6)	0.32	0.39
**Senility (R54)**	155	6.0	2.9 (2.5-3.4)	2.9 (2.2-3.7)	2.8 (2.2-3.5)	4.6 (3.0-6.7)	0.86	0.36
**Syncope and collapse (R55)**	133	5.1	2.9 (2.4-3.4)	3.0 (2.3-3.9)	2.8 (2.2-3.6)	4.6 (2.9-6.9)	0.77	0.4
**Other cataract (H26)**	132	5.1	0.6 (0.5-0.7)	0.5 (0.4-0.7)	0.6 (0.5-0.8)	0.6 (0.3-1.0)	0.05	0.58

For peer review only

**Unspecified acute lower respiratory infection (J22)**	103	4.0	1.8 (1.5-2.2)	1.4 (1.0-2.0)	1.9 (1.5-2.5)	3.9 (2.5-5.8)	1.24	0.21
**Cerebral infarction (I63)**	100	3.9	1.7 (1.4-2.1)	1.4 (1.0-2.0)	1.8 (1.3-2.3)	3.4 (2.1-5.2)	1.00	0.23
**Other symptoms & signs involving cognitive function and awareness (R41)**	96	3.7	5.2 (4.2-6.3)	4.2 (2.9-6.1)	5.9 (4.5-7.7)	8.1 (4.6-13.2)	1.93	0.05
**Pain in throat and chest (R07)**	91	3.5	1.4 (1.1-1.7)	1.7 (1.2-2.3)	1.3 (1.0-1.8)	1.2 (0.5-2.4)	-0.24	0.16
**Other chronic obstructive pulmonary disease (J44)**	87	3.4	1.0 (0.8-1.2)	1.0 (0.7-1.4)	1.0 (0.8-1.4)	0.8 (0.3-1.6)	-0.13	0.32
**Open wound of head (S01)**	78	3.0	3.0 (2.4-3.8)	3.2 (2.1-4.5)	2.8 (2.0-3.9)	4.5 (2.4-7.7)	0.65	0.47
**Unspecified dementia (F03)**	77	3.0	8.3 (6.6-10.4)	5.0 (2.9-7.9)	10.6 (7.9-14.0)	12.7 (6.8-21.7)	3.87	0.17
**Superficial injury of head (S00)**	73	2.8	3.6 (2.8-4.5)	3.6 (2.4-5.3)	3.3 (2.3-4.5)	6.9 (3.9-11.2)	1.64	0.39
**Heart failure (I50)**	61	2.3	0.8 (0.6-1.1)	0.6 (0.4-1.0)	1.0 (0.7-1.4)	0.8 (0.3-1.7)	0.08	0.76

For peer review only - http://bmjopen.bmj.com/site/about/guidelines.xhtml

**Acute renal failure (N17)**	61	2.3	2.1<br>(1.6-2.8)	1.6<br>(1.0-2.6)	2.2<br>(1.5-3.1)	5.7<br>(3.3-9.2)	2.05	0.25
**Alzheimer's disease (G30)**	60	2.3	10.4<br>(8.0-13.4)	7.0<br>(3.9-11.5)	10.8<br>(7.4-15.2)	31.9<br>(19.5-49.2)	12.46	0.24
**Other functional intestinal disorders (K59)**	59	2.3	1.8<br>(1.4-2.3)	2.6<br>(1.8-3.6)	1.5<br>(1.0-2.2)	0.9<br>(0.2-2.5)	-0.85	0.09
**Pneumonitis due to solids and liquids (J69)**	52	2.0	3.0<br>(2.2-3.9)	1.7<br>(0.8-3.0)	3.5<br>(2.4-5.0)	7.9<br>(4.4-13.1)	3.14	0.15

*The regression coefficient indicates the increase or decrease in standardised admission ratios associated with a one-step increase in severity of dementia. See Statistical analyses section for the linear model relating these variables.

CI, confidence interval

For peer review only

Table 3: Standardised admissions ratios (95% CI) by discharge diagnosis (ICD-10 chapter) for hospitalisations in the 12 month period after a dementia diagnosis

Discharge diagnosis by ICD-10 chapter		Number of hospitalisations with this discharge diagnosis	Proportion (%) of episodes with this discharge diagnosis	Standardised admission ratio (95% CI) by dementia severity at diagnosis				Regression Coefficient*	P>|t|*
				All cases	Mild N=986	Moderate N=1,328	Severe N=282		
**A**	Infectious and parasitic diseases	62	2.4	1.5 (1.2-2.0)	1.0 (0.6-1.7)	1.8 (1.3-2.5)	2.1 (0.9-3.9)	0.51	0.16
**B**	Infectious and parasitic diseases	11	0.4	1.6 (0.8-2.9)	0.8 (0.1-2.8)	2.1 (0.8-4.2)	2.8 (0.3-10.0)	0.99	0.11
**C**	Cancers	167	6.4	0.3 (0.3-0.4)	0.3 (0.3-0.4)	0.4 (0.3-0.5)	0.1 (0.1-0.3)	-0.1	0.47
**D**	Benign neoplasms or diseases of the blood	158	6.1	0.8 (0.7-1.0)	0.9 (0.7-1.1)	0.9 (0.7-1.1)	0.3 (0.1-0.7)	-0.3	0.31
**E**	Endocrine, nutritional and metabolic diseases	138	5.3	1.9 (1.6-2.3)	1.9 (1.5-2.5)	1.9 (1.4-2.4)	2.1 (1.2-3.5)	0.09	0.54
**F**	Mental and behavioural disorders	194	7.5	5.7 (4.9-6.6)	4.7 (3.6-6.1)	6.1 (5-7.4)	7.4 (4.9-10.8)	1.35	0.01
**G**	Diseases of the nervous system	183	7.0	2.4 (2.1-2.8)	2.1 (1.6-2.7)	2.4 (2.0-3.0)	3.4 (2.3-5.0)	0.65	0.18
**H**	Diseases of the eye	209	8.1	0.6	0.6	0.7	0.3	-0.13	0.43

For peer review only - http://bmjopen.bmj.com/site/about/guidelines.xhtml

				(0.5-0.7)	(0.5-0.8)	(0.5-0.8)	(0.2-0.6)		
**I**	Diseases of the circulatory system	638	24.6	1.3 (1.2-1.3)	1.4 (1.2-1.5)	1.2 (1.1-1.4)	1.0 (0.8-1.4)	-0.16	0.06
**J**	Diseases of the respiratory system	567	21.8	1.6 (1.5-1.6)	1.3 (1.1-1.5)	1.7 (1.5-1.9)	2.2 (1.8-2.7)	0.46	0.03
**K**	Diseases of the digestive system	448	17.3	1.0 (0.9-1.0)	1.2 (1.1-1.4)	1.0 (0.8-1.1)	0.6 (0.4-0.9)	-0.3	0.06
**L**	Diseases of the skin	105	4.0	1.2 (1.0-1.5)	1.2 (0.9-1.6)	1.1 (0.8-1.4)	1.9 (1.1-3.0)	0.33	0.41
**M**	Diseases of the musculoskeletal system	239	9.2	0.9 (0.8-1.1)	1.1 (0.9-1.3)	0.8 (0.7-1.0)	0.9 (0.6-1.4)	-0.07	0.64
**N**	Diseases of the genitourinary system	1389	53.5	1.3 (1.2-1.3)	1.1 (1.0-1.1)	1.4 (1.3-1.4)	1.7 (1.4-1.9)	0.28	0.01
**R**	Symptoms and sign not elsewhere classified	913	35.2	1.9 (1.8-1.9)	2 (1.8-2.2)	1.9 (1.7-1.9)	1.7 (1.3-2.1)	-0.15	0.18
**S**	Injury, poisoning and certain other external causes	574	22.1	2.3 (2.1-2.3)	2.3 (2.0-2.7)	2.2 (2.0-2.5)	2.4 (1.9-3.1)	0.05	0.69
**T**	Injury, poisoning and certain other external causes	120	4.6	1.6 (1.3-1.9)	1.6 (1.2-2.1)	1.8 (1.4-2.3)	0.7 (0.2-1.5)	-0.46	0.47
**Z**	External causes	161	6.2	1.1	1.6	0.9	0.8	-0.4	0.25

For peer review only - http://bmjopen.bmj.com/site/about/guidelines.xhtml

	(1.0-1.3)	(1.3-1.9)	(0.7-1.1)	(0.4-1.4)	

*The regression coefficient indicates the increase or decrease in SAR associated with a one-step increase in severity of dementia. See Statistical analyses section for the linear model relating these variables.

CI, confidence interval

For peer review only

For peer review only - http://bmjopen.bmj.com/site/about/guidelines.xhtml

SARs for each discharge diagnosis by the severity of dementia at diagnosis are shown in Tables 2 and 3. For the cohort as a whole, patients with dementia were hospitalised more than the catchment population (SAR significantly greater than 1) for most of the 20 most common discharge diagnoses, the exceptions being chronic renal failure (N18), other chronic obstructive pulmonary disease (J44), heart failure (I50) (SARs not significantly >1), and other cataracts (H26, SAR <1). The trends across dementia severity groups were positive for 17 of the 20 conditions (increased SARs with increasing severity, indicated by a positive regression coefficient); however, only the trend for the R41 code ("other symptoms and signs involving cognitive function and awareness") was statistically significant. Considering ICD chapters, in most groups, patients with dementia were hospitalised more than the catchment population (Table 3). The exceptions were cancers, benign neoplasms and diseases of the eye (SAR < 1). Of the 18 ICD-10 chapters, trends across dementia severity groups were positive in ten; however, only those for mental and behavioural disorders (F), diseases of the respiratory system (J), and diseases of the genitourinary system (N) were statistically significant. No chapters showed significantly decreasing SARs with increasing dementia severity, although the negative trend for diseases of the digestive system (K) was close to significance. After excluding repeat admissions for the same cause, similar trends were observed at the 3-digit ICD and chapter level, although reached or approached statistical significance for disorders of the urinary system and pneumonia with an increasing trend with dementia severity, and a decreasing trend for pain in throat and chest (Tables S3-4, Figures S1-2).

For peer review only

DISCUSSION

In this retrospective cohort study of people with a dementia diagnosis, over half had at least one hospitalisation in the 12-months after diagnosis. The risk of hospitalisation and the length of hospital stay increased with higher dementia severity. Most of the common reasons for hospitalisation, based on the primary discharge diagnosis, were also more common than expected in cases relative to the catchment population. Differences in SARs by baseline dementia severity were more evident for discharge diagnoses grouped at ICD-10-chapter level than at the level of specific 3-digit ICD-10 codes.

The hospitalisation rate for at least one inpatient admission in the 12-month period after dementia diagnosis was 58 per 100 py, slightly higher than reported elsewhere.[12, 13] This may reflect more recent data (given that hospitalisation rates are increasing), the near-complete data on hospitalisation in our study, and/or the fact that hospitalisation is free at the point of delivery in the UK context.[14] Increasing likelihood and LOS with increasing dementia severity is consistent with other reports, although there are differences between admissions from community and institutional facilities, with reduced healthcare use following relocation to institutional care, potentially reflecting higher support.[15-17] This, as well as survival effects, may account for our finding that the most marked difference in hospitalisation was between mild and moderate dementia rather than between moderate and severe dementia.

The most common causes of hospital admission, after excluding repeat admissions for the same cause, were disorders of the urinary system and pneumonia, followed by fracture of femur, senility and syncope and collapse; this is similar to findings reported elsewhere.[3, 18-20] Most

For peer review only - http://bmjopen.bmj.com/site/about/guidelines.xhtml

of the common causes of admission identified in people with dementia were more frequent than expected relative to the catchment population (SAR > 1).

Higher risk of syncope and collapse, and of other diagnoses related to fractures and falls, have been previously reported in patients with dementia,[3, 18] and may reflect age-related autonomic dysfunction, comorbid disorders and polypharmacy,[21] impaired visuospatial functions and gait instability.[22] A review of published clinical trials of atypical anti-psychotic medication use did not find evidence of increased injury, falls, or syncope associated with their use,[23] nor did a recent study of hospitalised falls/fractures in a CRIS-derived cohort of people with dementia.[24] Significant predictors included social/demographic factors, physical health problems, and previous episode; no associations were found with neuropsychiatric symptoms, cognitive (MMSE) scores, or functional problems.[24] Inpatient admissions due to infection were also more common than expected: both at the level of specific disorders and at the ICD-10-chapter level. This may reflect more rapid age-related decline in immune function,[25] problems with mobility associated with urinary incontinence and subsequent infection,[26] or inactivity and dysphagia causing respiratory tract infection.[27]

A lower than expected frequency of hospitalisation (SAR <1) was observed for heart failure, cancers, benign neoplasms and diseases of the eye, consistent with other reports,[12, 20] and this may reflect diagnostic delay. For example, an association of dementia with later-stage cancer at diagnosis has been reported,[28] as well as unexpected cases being identified at autopsy;[29] furthermore, patients with dementia may be less likely to undergo invasive diagnostic tests and receive fewer treatment interventions, potentially explaining a shorter duration of survival.[30]

For peer review only - http://bmjopen.bmj.com/site/about/guidelines.xhtml

While rates of healthcare utilisation and costs, or limited specified causes of admission,[15, 16] have been reported in relation to severity of dementia, we are not aware of any evaluation of common causes of hospital admission by dementia stage. Most of the SARs for specific causes of hospitalisation were positively associated with dementia severity, although most not to a significant extent. Associations, where significant, were more often with discharge diagnoses categorised by ICD-10 chapter rather than 3-character code, most likely because of higher statistical power. As well as hospitalisations as a direct result of dementia, with worsening cognitive impairment, there may be an increase in admissions to acute care for exacerbation, due to an individual's decreasing ability to manage existing comorbidity. Similarly, if identification of new comorbidity is delayed at earlier stages of dementia, this may only come to light when it is sufficiently advanced to require hospitalisation. Increasing respiratory and genitourinary disease admissions most likely represent infections, and diseases of the nervous system may reflect dementia being provided as a primary diagnosis, or else comorbidities such as depression, psychosis or other behavioural/psychological manifestations. Risk for diseases of the digestive system diminished across the severity groups, but this may reflect a diminished ability to communicate non-specific symptoms rather than a reduced risk of defined disorders, as indicated by the negative coefficient for 'other functional intestinal disorders' (K59) in Table 2, which was the most common digestive system diagnosis.

For peer review only

Strengths of the study included the large and representative sample of diagnosed cases of dementia and the linkage to a national hospitalisation database providing near-complete outcome ascertainment. Patients who were active to acute hospital liaison services at the time of their dementia diagnosis were excluded from the study since these may reflect individuals whose diagnosis might have been precipitated by the hospitalisation, and yet who would also have an increased risk of re-hospitalisation by virtue of their status, thus biasing the association

For peer review only - http://bmjopen.bmj.com/site/about/guidelines.xhtml

of interest. Considering limitations, the analysed sample was from a single service and also only included cases with dementia diagnosed in specialist services; however, estimated proportions of people with dementia in the SLaM catchment who receive a specialist diagnosis is relatively high at 75.2%, compared to 67.6% nationally.[31] Furthermore, unrecognised dementia cases omitted from observed admission rates will have biased findings towards the null, as would healthy survivor effects. We used the discharge diagnosis recorded during the last episode of the hospital spell, and it is possible that the primary discharge diagnosis code may reflect complications that arose during the hospital stay rather than the reason for initial hospital admission. Finally, this analysis did not attempt to account for comorbidity, medication use, institutional residence or socioeconomic status, and did not sub-classify dementia.

Understanding the factors influencing health service utilisation by patients with dementia is necessary to inform care needs and to guide future healthcare resource planning and allocation. Our findings are of importance given that hospitalisation is a significant element in the cost of dementia care and that the prevalence of dementia is increasing. Our study highlights the need to develop specific strategies for those causes of hospitalisation that are most amenable to prevention in dementia. Further research on factors influencing patterns of healthcare use over time and severity would be useful in this context.

For peer review only

Funding

This work was supported by NIHR Biomedical Research Centre at the South London and Maudsley NHS Foundation Trust and King's College London. RS is additionally part-funded by a Medical Research Council Mental Health Data Pathfinder Award to King's College London, and an NIHR Senior Investigator Award

Role of the funding source

The funder of the study had no role in the study design, data collection, data analysis, data interpretation, or writing of the report. UG, GP and RS had access to the anonymised data. The corresponding author had the final responsibility for the decision to submit for publication.

Declaration of Competing Interest

UG and NG are employees of GSK, hold stock and receive a salary from GSK. RS has received research funding in the last 5 years from Roche, Janssen, Takeda and GSK.

Acknowledgments

The authors would like to acknowledge Hitesh Shetty for his contributions to the study.

Data sharing

Because of their nature and to comply with their ethical approval, CRIS data are required to remain within the firewall of the South London and Maudsley NHS Foundation Trust (SLaM). Access to the data used for this study can be facilitated by the CRIS Oversight Committee on application and with appropriate SLaM affiliation, details of which can be obtained from cris.administrator@slam.nhs.uk.

For peer review only

For peer review only - http://bmjopen.bmj.com/site/about/guidelines.xhtml

References:

1. Alzheimer's_Society. Dementia UK Update, 2014.

2. Phelan EA, Borson S, Grothaus L, et al. Association of incident dementia with hospitalizations. *JAMA* 2012;307(2):165–72.

3. Natalwala A, Potluri R, Uppal H, et al. Reasons for hospital admissions in dementia patients in Birmingham, UK, during 2002-2007. *Dement Geriatr Cogn Disord* 2008;26(6):499–505.

4. Mukadam N, Sampson EL. A systematic review of the prevalence, associations and outcomes of dementia in older general hospital inpatients. *Int Psychogeriatr* 2011;23(3):344–55.

5. Lehmann J, Michalowsky B, Kaczynski A, et al. The Impact of Hospitalization on Readmission, Institutionalization, and Mortality of People with Dementia: A Systematic Review and Meta-Analysis. *J Alzheimers Dis* 2018;64(3):735–49.

6. Perera G, Broadbent M, Callard F, et al. Cohort profile of the South London and Maudsley NHS Foundation Trust Biomedical Research Centre (SLaM BRC) Case Register: current status and recent enhancement of an Electronic Mental Health Record-derived data resource. *BMJ Open* 2016;6(3):e008721.

7. Jackson RG, Patel R, Jayatilleke N, et al. Natural language processing to extract symptoms of severe mental illness from clinical text: the Clinical Record Interactive Search Comprehensive Data Extraction (CRIS-CODE) project. *BMJ Open* 2017;7(1):e012012.

8. NHS. Hospital Episode Statistics (HES). Secondary Hospital Episode Statistics (HES) 2019. https://digital.nhs.uk/data-and-information/data-tools-and-services/data-services/hospital-episode-statistics. (accessed 13-Jan-2019)

9. Pirkis JE, Burgess PM, Kirk PK, et al. A review of the psychometric properties of the Health of the Nation Outcome Scales (HoNOS) family of measures. *Health Qual Life Outcomes* 2005;3(1):76.

For peer review only - http://bmjopen.bmj.com/site/about/guidelines.xhtml

10. Canuto A, Weber K, Gold G, et al. Structured assessment of mental health status in psychogeriatrics: validity of the French HoNOS65+. *Can J Psychiatry* 2007;52(1):37–45.

11. NICE. Donepezil, galantamine, rivastigmine and memantine for the treatment of dementia. Health technology appraisal 2018 update. In: NICE, ed. 2018 ed, 2018.

12. Tuppin P, Kusnik-Joinville O, Weill A, et al. Primary health care use and reasons for hospital admissions in dementia patients in france: database study for 2007. *Dement Geriatr Cogn Disord* 2009;28(3):225–32.

13. Chen L, Reed C, Happich M, et al. Health care resource utilisation in primary care prior to and after a diagnosis of Alzheimer's disease: a retrospective, matched case-control study in the United Kingdom. *BMC Geriatr* 2014;14:76.

14. Bernardes C, Massano J, Freitas A. Hospital admissions 2000-2014: A retrospective analysis of 288 096 events in patients with dementia. *Arch Gerontol Geriatr* 2018;77:150–57.

15. Lin PJ, Rane PB, Fillit HM, et al. National estimates of potentially avoidable hospitalizations among medicare beneficiaries with Alzheimer's disease and related dementias. *Alzheimer's and Dementia* 2016;12(7):P253–P54.

16. Zhu CW, Cosentino S, Ornstein K, et al. Use and cost of hospitalization in dementia: Longitudinal results from a community-based study. *Int J Geriatr Psychiatry* 2015;30(8):833–41.

17. Fillenbaum G, Heyman A, Peterson BL, et al. Use and cost of hospitalization of patients with AD by stage and living arrangement: CERAD XXI. *Neurology* 2001;56(2):201–6.

18. Zhao Yang Y. Healthcare costs and utilization for Medicare beneficiaries with Alzheimer's. *BMC Health Serv Res* 2008;8(108).

19. Rao A, Suliman A, Vuik S, et al. Outcomes of dementia: Systematic review and meta-analysis of hospital administrative database studies. *Arch Gerontol Geriatr* 2016;66:198–204.

For peer review only - http://bmjopen.bmj.com/site/about/guidelines.xhtml

20. Guijarro R, San Roman CM, Gomez-Huelgas R, et al. Impact of dementia on hospitalization. *Neuroepidemiology* 2010;35(2):101–8.

21. O' Brien H, Kenny A. Syncope in the Elderly. *Eur Cardiol* 2014;9(1):28–36.

22. Sheridan PL, Hausdorff JM. The role of higher-level cognitive function in gait: executive dysfunction contributes to fall risk in Alzheimer's disease. *Dement Geriatr Cogn Disord* 2007;24(2):125–37.

23. Schneider LS, Dagerman K, Insel PS. Efficacy and adverse effects of atypical antipsychotics for dementia: meta-analysis of randomized, placebo-controlled trials. *Am J Geriatr Psychiatry* 2006;14(3):191–210.

24. Sharma S, Mueller C, Stewart R, et al. Predictors of Falls and Fractures Leading to Hospitalization in People With Dementia: A Representative Cohort Study. *J Am Med Dir Assoc* 2018;19(7):607–12.

25. Janssens JP. Pneumonia in the elderly (geriatric) population. *Curr Opin Pulm Med* 2005;11(3):226–30.

26. Schnelle JF, Leung FW. Urinary and fecal incontinence in nursing homes. *Gastroenterology* 2004;126(1 Suppl 1):S41–7.

27. Palmer JL, Metheny NA. Preventing aspiration in older adults with dysphagia. *Am J Nurs* 2008;108(2):40–8; quiz 49.

28. Gorin SS, Heck JE, Albert S, et al. Treatment for breast cancer in patients with Alzheimer's disease. *J Am Geriatr Soc* 2005;53(11):1897–904.

29. Gupta SK, Lamont EB. Patterns of presentation, diagnosis, and treatment in older patients with colon cancer and comorbid dementia. *J Am Geriatr Soc* 2004;52(10):1681–7.

30. Libert Y, Dubruille S, Borghgraef C, et al. Vulnerabilities in Older Patients when Cancer Treatment is Initiated: Does a Cognitive Impairment Impact the Two-Year Survival? *PLoS One* 2016;11(8):e0159734.

For peer review only

For peer review only - http://bmjopen.bmj.com/site/about/guidelines.xhtml

31. NHS England. Dementia diagnosis monthly workbook. Secondary Dementia diagnosis monthly workbook 2017. https://www.england.nhs.uk/mental-health/dementia/monthly-workbook/. (accessed 30-Mar-2017)

For peer review only

For peer review only - http://bmjopen.bmj.com/site/about/guidelines.xhtml

Table S1: **Twenty most common primary discharge diagnoses amongst individuals with dementia receiving inpatient care in the 12 months after dementia diagnosis (repeat admissions excluded)**

Primary discharge diagnosis (ICD code)*	Number of episodes	% of total inpatient care episodes
Other disorders of urinary system (N39)	399	15.4
Pneumonia, organism unspecified (J18)	248	9.6
Fracture of femur (S72)	150	5.8
Senility (R54)	128	4.9
Syncope and collapse (R55)	117	4.5
Other cataract (H26)	107	4.1
Unspecified acute lower respiratory infection (J22)	97	3.7
Cerebral infarction (I63)	86	3.3
Other symptoms & signs involving cognitive function and awareness (R41)	86	3.3
Pain in throat and chest (R07)	78	3.0
Open wound of head (S01)	75	2.9
Unspecified dementia (F03)	73	2.8
Other chronic obstructive pulmonary disease (J44)	71	2.7
Superficial injury of head (S00)	67	2.6
Acute renal failure (N17)	57	2.2
Alzheimer's disease (G30)	56	2.2
Heart failure (I50)	52	2.0
Pneumonitis due to solids and liquids (J69)	49	1.9
Other joint disorders, not elsewhere classified (M25)	46	1.8
Other functional intestinal disorders (K59)	43	1.7

*Three digit ICD-10 discharge diagnoses; repeat admissions for the same diagnosis removed. Percentages based on 2,596 individuals with at least 1 inpatient admission in the 12 month period after the dementia diagnosis date

For peer review only

Table S2: **ICD chapter level primary discharge diagnoses amongst individuals with dementia receiving inpatient care in the 12 months after dementia diagnosis (repeat episodes excluded)***

Chapter		Number of episodes	% of total inpatient care episodes
**A**	Infectious and parasitic diseases	59	2.3
**B**	Infectious and parasitic diseases	11	0.4
**C**	Cancers	123	4.7
**D**	Benign neoplasms or diseases of the blood	119	4.6
**E**	Endocrine, nutritional and metabolic diseases	122	4.7
**F**	Mental and behavioural disorders	180	6.9
**G**	Diseases of the nervous system	159	6.1
**H**	Diseases of the eye	166	6.4
**I**	Diseases of the circulatory system	542	20.9
**J**	Diseases of the respiratory system	514	19.8
**K**	Diseases of the digestive system	395	15.2
**L**	Diseases of the skin	89	3.4
**M**	Diseases of the musculoskeletal system	214	8.2
**N**	Diseases of the genitourinary system	554	21.3
**R**	Symptoms and signs not elsewhere classified	803	30.9
**S**	Injury, poisoning and certain other external causes	545	21.0
**T**	Injury, poisoning and certain other external causes	100	3.9
**Z**	External causes	140	5.4

*Percentages based on 2,596 individuals with at least 1 inpatient admission in the 12 month period after the dementia diagnosis date. Repeat episodes for the same 3-digit ICD code in the 12-month time period excluded

For peer review only

For peer review only - http://bmjopen.bmj.com/site/about/guidelines.xhtml

Table S3: Standardised admissions ratios (95% CI) for hospitalisations in the 12 months following a first dementia diagnosis – by 3 digit ICD-10 code for the primary discharge diagnosis* (repeat admissions excluded)

Primary discharge diagnosis	By dementia severity at first diagnosis					
	All	Mild	Moderate	Severe	Coefficient†	P-value†
Other disorders of urinary system (N39)	2.5 (2.3-2.5)	2.3 (2.0-2.7)	2.6 (2.3-3.0)	2.9 (2.2-3.8)	0.29	**0.01**
Pneumonia, organism unspecified (J18)	2.0 (1.7-2.3)	1.5 (1.2-1.9)	2.1 (1.8-2.5)	3.0 (2.2-4.1)	0.77	0.07
Fracture of femur (S72)	2.4 (2.0-2.8)	2.4 (1.8-3.2)	2.4 (1.9-3.0)	2.5 (1.4-3.9)	0.01	0.8
Senility (R54)	2.8 (2.4-3.4)	2.8 (2.0-3.7)	2.8 (2.1-3.5)	3.4 (2.0-5.5)	0.32	0.33
Syncope and collapse (R55)	2.8 (2.3-3.4)	2.8 (2.1-3.8)	2.8 (2.1-3.6)	3.0 (1.6-5.1)	0.07	0.44
Other cataract (H26)	0.6 (0.5-0.7)	0.5 (0.3-0.7)	0.7 (0.5-0.9)	0.5 (0.3-1.0)	0.04	0.8
Unspecified acute lower respiratory infection (J22)	1.9 (1.5-2.3)	1.5 (1.0-2.1)	1.9 (1.4-2.5)	2.8 (1.6-4.6)	0.66	0.12
Cerebral infarction (I63)	1.7 (1.3-2.1)	1.4 (0.9-2.1)	1.6 (1.2-2.2)	2.7 (1.5-4.5)	0.65	0.24
Other symptoms & signs involving cognitive function and awareness (R41)	5.1 (4.0-6.2)	4.2 (2.7-6.1)	5.7 (4.3-7.6)	4.9 (2.3-9.4)	0.39	0.67
Pain in throat and chest (R07)	1.4 (1.1-1.7)	1.7 (1.2-2.3)	1.2 (0.9-1.7)	0.8 (0.3-2.0)	-0.42	**0.03**
Open wound of head (S01)	3.0 (2.4-3.7)	3.3 (2.2-4.7)	2.7 (1.9-3.8)	3.2 (1.5-6.1)	-0.04	0.91
Unspecified dementia (F03)	8.6 (6.7-10.8)	5.4 (3.1-8.6)	10.7 (7.9-14.2)	9.6 (4.4-18.3)	2.11	0.46
Other chronic obstructive pulmonary disease (J44)	1.1 (0.8-1.4)	1.1 (0.7-1.6)	1.1 (0.8-1.5)	0.7 (0.2-1.7)	-0.18	0.35
Superficial injury of head (S00)	3.4 (2.6-4.3)	3.3 (2.1-5.0)	3.1 (2.1-4.4)	4.9 (2.5-8.8)	0.79	0.41
Acute renal failure (N17)	2.2 (1.7-2.9)	1.6 (0.9-2.6)	2.4 (1.6-3.3)	3.7 (1.8-6.8)	1.05	0.1
Alzheimer's disease (G30)	10.4 (7.9-13.6)	7.5 (4.2-12.3)	10.5 (7.0-15.0)	20.5 (10.6-35.8)	6.5	0.19
Heart failure (I50)	0.9 (0.6-1.1)	0.6 (0.4-1.1)	1.1 (0.7-1.5)	0.6 (0.2-1.6)	-0.02	0.96
Pneumonitis due to solids and liquids (J69)	3.0 (2.2-4.0)	1.6 (0.8-3.0)	3.7 (2.5-5.2)	4.6 (2.0-9.0)	1.46	0.14
Other joint disorders, not elsewhere classified (M25)	1.7 (1.3-2.3)	1.3 (0.7-2.2)	1.8 (1.2-2.7)	2.8 (1.2-5.6)	0.79	0.09
Other functional intestinal disorders (K59)	1.5 (1.1-2.0)	1.8 (1.1-2.8)	1.4 (0.9-2.2)	0.7 (0.1-2.4)	-0.58	0.12

*Based on 2,617 individuals with at least one hospitalisation in the pre index period and 2,596 individuals in the post index period

†The regression coefficient indicates the increase or decrease in SAR associated with a one-step increase in severity of dementia. See Statistical analyses section for the linear model relating these variables.

For peer review only - http://bmjopen.bmj.com/site/about/guidelines.xhtml

For peer review only

Table S4: Standardised admissions ratios (95% CI) by ICD chapters (repeat admissions excluded)[*]

	Chapter	All	Mild	Moderate	Severe	Coefficient[†]	p-value †
**A**	Infectious and parasitic diseases	1.6 (1.2-2.0)	1.0 (0.6-1.7)	1.8 (1.3-2.5)	2.2 (1.0-4.2)	0.58	0.11
**B**	Infectious and parasitic diseases	1.7 (0.9-3.1)	0.8 (0.1-3.0)	2.2 (0.9-4.5)	2.9 (0.4-10.6)	1.06	0.11
**C**	Cancers	0.6 (0.5-0.7)	0.7 (0.5-0.8)	0.6 (0.4-0.7)	0.3 (0.1-0.7)	-0.17	0.19
**D**	Benign neoplasms or diseases of the blood	1.0 (0.8-1.2)	1.1 (0.9-1.5)	1.0 (0.8-1.3)	0.5 (0.2-1.1)	-0.32	0.22
**E**	Endocrine, nutritional and metabolic diseases	2.0 (1.7-2.4)	2.0 (1.5-2.7)	1.9 (1.5-2.5)	2.5 (1.4-4.1)	0.27	0.39
**F**	Mental and behavioural disorders	5.8 (4.9-6.7)	5.0 (3.8-6.4)	6.2 (5.0-7.5)	6.6 (4.1-9.9)	0.8	0.19
**G**	Diseases of the nervous system	2.5 (2.1-2.9)	2.3 (1.7-2.9)	2.6 (2.1-3.3)	3.1 (1.9-4.7)	0.4	0.03
**H**	Diseases of the eye	0.6 (0.6-0.8)	0.6 (0.5-0.8)	0.7 (0.6-0.9)	0.5 (0.2-0.8)	-0.07	0.65
**I**	Diseases of the circulatory system	1.2 (1.1-1.2)	1.3 (1.1-1.5)	1.2 (1.1-1.4)	1.1 (0.8-1.4)	-0.11	0.08
**J**	Diseases of the respiratory system	1.7 (1.5-1.7)	1.3 (1.1-1.6)	1.8 (1.6-2.0)	2.2 (1.8-2.8)	0.45	0.03
**K**	Diseases of the digestive system	1.0 (0.9-1.0)	1.1 (1.0-1.3)	0.9 (0.8-1.1)	0.7 (0.4-1.0)	-0.25	0.06
**L**	Diseases of the skin	1.1 (0.9-1.4)	1.1 (0.8-1.5)	1.0 (0.7-1.4)	1.8 (1.0-2.9)	0.34	0.42
**M**	Diseases of the musculoskeletal system	0.9 (0.8-1.1)	1.0 (0.8-1.3)	0.9 (0.7-1.1)	1.0 (0.7-1.5)	-0.01	0.95
**N**	Diseases of the genitourinary system	1.8 (1.7-1.8)	1.6 (1.4-1.9)	1.9 (1.7-2.1)	2.2 (1.7-2.8)	0.28	0.01
**R**	Symptoms and sign not elsewhere classified	1.9 (1.7-1.9)	1.9 (1.7-2.1)	1.9 (1.7-1.9)	1.7 (1.3-2.1)	-0.12	0.29
**S**	Injury, poisoning and certain other external causes	2.3 (2.1-2.3)	2.3 (2.0-2.7)	2.2 (1.9-2.4)	2.4 (1.8-3.1)	0.03	0.86
**T**	Injury, poisoning and certain other external causes	1.5 (1.3-1.9)	1.5 (1.1-2.1)	1.8 (1.3-2.3)	0.7 (0.2-1.7)	-0.37	0.49
**Z**	External causes	1.1 (0.9-1.3)	1.5 (1.2-1.9)	0.9 (0.7-1.2)	0.8 (0.4-1.4)	-0.34	0.22
Total		1.4 (1.4-1.4)	1.4 (1.3-1.4)	1.4 (1.4-1.4)	1.4 (1.3-1.4)	0.01	0.49

[*]Based on 2,617 individuals with at least one hospitalisation in the pre index period and 2,596 individuals in the post index period [†]The regression coefficient indicates the increase or decrease in SAR associated with a one-step increase in severity of dementia. See Statistical analyses section for the linear model relating these variables.

For peer review only - http://bmjopen.bmj.com/site/about/guidelines.xhtml

Figure S1: Standardised admissions ratios by 3-digit ICD code for mild, moderate and severe dementia populations (excludes repeat admissions for the same cause at a 3-digit ICD level)

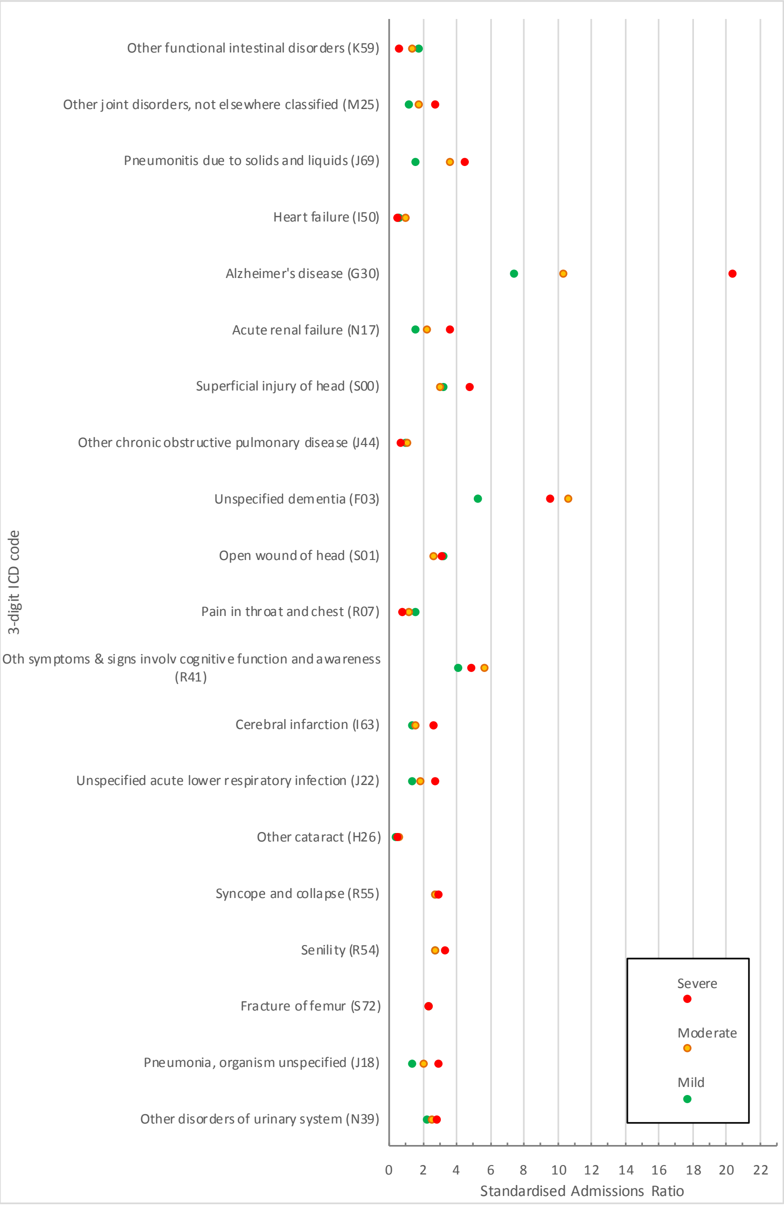

For peer review only - http://bmjopen.bmj.com/site/about/guidelines.xhtml

Figure S2: Standardised admissions ratios by ICD chapter for mild, moderate and severe dementia populations (excludes repeat admissions for the same cause at a 3-digit ICD level)

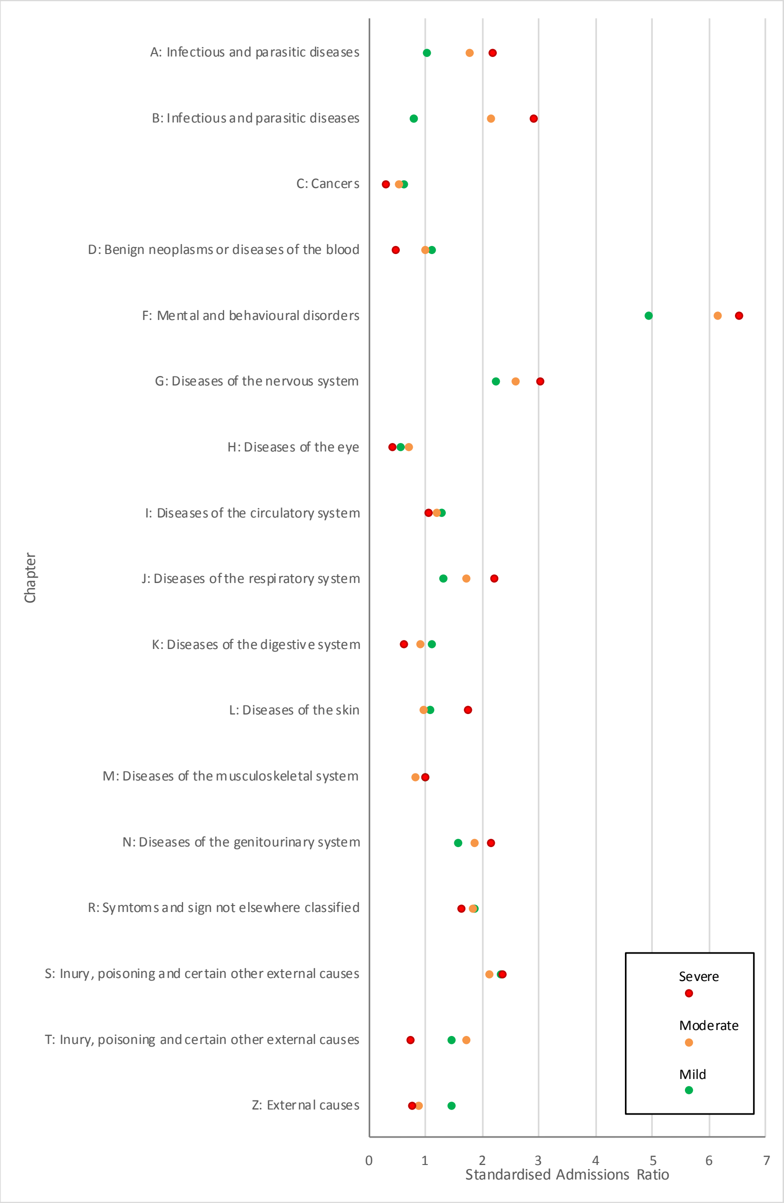

For peer review only - http://bmjopen.bmj.com/site/about/guidelines.xhtml

The association between dementia severity and hospitalisation profile in a newly assessed clinical cohort: The South London and Maudsley Case Register

Journal:	*BMJ Open*
Manuscript ID	bmjopen-2019-035779.R1
Article Type:	Original research
Date Submitted by the Author:	07-Feb-2020
Complete List of Authors:	Gungabissoon, Usha; GSK, Epidemiology (Value Evidence and Outcomes)<br>Perera, Gayan; King's College London - Institute of Psychiatry, Psychological Medicine<br>Galwey, Nicholas; GSK, Target Sciences<br>Stewart, Robert; South London and Maudsley NHS Foundation Trust
**Primary Subject Heading**:	Epidemiology
Secondary Subject Heading:	Geriatric medicine, Health policy
Keywords:	Dementia < NEUROLOGY, EPIDEMIOLOGY, HEALTH ECONOMICS

I, the Submitting Author has the right to grant and does grant on behalf of all authors of the Work (as defined in the below author licence), an exclusive licence and/or a non-exclusive licence for contributions from authors who are: i) UK Crown employees; ii) where BMJ has agreed a CC-BY licence shall apply, and/or iii) in accordance with the terms applicable for US Federal Government officers or employees acting as part of their official duties; on a worldwide, perpetual, irrevocable, royalty-free basis to BMJ Publishing Group Ltd ("BMJ") its licensees and where the relevant Journal is co-owned by BMJ to the co-owners of the Journal, to publish the Work in this journal and any other BMJ products and to exploit all rights, as set out in our licence.

The Submitting Author accepts and understands that any supply made under these terms is made by BMJ to the Submitting Author unless you are acting as an employee on behalf of your employer or a postgraduate student of an affiliated institution which is paying any applicable article publishing charge ("APC") for Open Access articles. Where the Submitting Author wishes to make the Work available on an Open Access basis (and intends to pay the relevant APC), the terms of reuse of such Open Access shall be governed by a Creative Commons licence – details of these licences and which Creative Commons licence will apply to this Work are set out in our licence referred to above.

Other than as permitted in any relevant BMJ Author's Self Archiving Policies, I confirm this Work has not been accepted for publication elsewhere, is not being considered for publication elsewhere and does not duplicate material already published. I confirm all authors consent to publication of this Work and authorise the granting of this licence.

For peer review only

The association between dementia severity and hospitalisation profile in a newly assessed clinical cohort: The South London and Maudsley Case Register

Authors:

Usha Gungabissoon[1,2], M.Sc.; Gayan Perera[1], Ph.D.; Nicholas Galwey[3], Ph.D.; Robert Stewart[1,4], M.D., FRCPsych

[1]Institute of Psychiatry, Psychology and Neuroscience (IOPPN), King's College London, London, United Kingdom (UK)

[2]Epidemiology, Value Evidence and Outcomes, Global Medical, GlaxoSmithKline (GSK) R&D, London, UK

[3]Target Sciences, GSK R&D, London, UK

[4]South London and Maudsley NHS Foundation Trust, London, UK

Corresponding author:

Usha Gungabissoon

Department of Psychological Medicine (PO84), Institute of Psychiatry, Psychology and Neuroscience, King's College London. De Crespigny Park, London SE5 8AF

Email: usha.2.gungabissoon@gsk.com

Phone: +442089902469

For peer review only

For peer review only - http://bmjopen.bmj.com/site/about/guidelines.xhtml

Word count (excluding title page, abstract, references, figures and tables): 3730 words

For peer review only

For peer review only - http://bmjopen.bmj.com/site/about/guidelines.xhtml

ABSTRACT

Objectives

To evaluate the risk and common causes of hospitalisation in patients with newly diagnosed dementia and variation by severity of cognitive impairment.

Setting

We used data from a large London mental healthcare case register linked to a national hospitalisation database.

Participants

Individuals aged ≥65 years with newly diagnosed dementia with recorded cognitive function and the catchment population within the same geography

Outcome measures

We evaluated the risk and duration of hospitalisation in the year following a dementia diagnosis. In addition we identified the most common causes of hospitalisation and calculated age- and gender-standardised admission ratios by dementia severity (mild/moderate/severe) relative to the catchment population.

Results

For peer review only

Of the 5,218 patients with dementia, 2,596 (49.8%) were hospitalised in the year following diagnosis. The proportion of individuals with mild, moderate and severe dementia that had a hospital admission was 47.9%, 50.8% and 51.7% respectively (p 0.097). Duration of hospital-stay increased with dementia severity (median 2 days in mild to 4 days in severe dementia, p 0.0001). After excluding re-admissions for the same cause, the most common primary hospitalisation discharge diagnoses amongst patients with dementia were urinary system disorders, pneumonia, and fracture of femur, accounting for 15%, 10% and 6% of admissions respectively. Overall, patients with dementia were hospitalised 30% more than the catchment population, and this trend was observed for most of the discharge diagnoses evaluated. Standardised admission ratios for urinary and respiratory disorders were higher in those with more severe dementia at diagnosis.

Conclusions

Individuals with a dementia diagnosis were more likely to be hospitalised than individuals in the catchment population. The length of hospital-stay increased with dementia severity. Most of the common causes of hospitalisation were more common than expected relative to the catchment population, but standardised admission ratios only varied by dementia stage for certain groups of conditions.

Key words: burden, dementia, epidemiology, healthcare utilisation, hospitalisation

For peer review only

For peer review only

For peer review only - http://bmjopen.bmj.com/site/about/guidelines.xhtml

STRENGTHS AND LIMITATIONS OF THIS STUDY

- We evaluated hospitalisation in a large and representative sample of newly diagnosed cases of dementia in a London mental healthcare case register linked to a national hospitalisation database providing near-complete outcome ascertainment.
- Severity of dementia at the time of diagnosis was determined from the Mini Mental State Examination score or Health of the Nation Outcome Scales impairment score, which has acceptable/good psychometric properties and correlates with MMSE measurement.
- We obtained the analysed samples from a single service provider in a single London catchment, albeit highly socially diverse, which may limit the generalisability of our findings to other settings.
- The analyses did not account for comorbidities, use of medications, institutional residence or socioeconomic status, aetiology of dementia or hospitalisation rates prior to the diagnosis of dementia, all of which may influence the risk of hospitalisation.

For peer review only - http://bmjopen.bmj.com/site/about/guidelines.xhtml

INTRODUCTION

In the United Kingdom (UK) there are around 850,000 people living with dementia and the associated annual healthcare costs are around £4.3 billion; major contributing factors to costs include disease severity and hospitalisation.[1] It follows that the associated healthcare costs are expected to increase in line with the increasing prevalence of dementia as a result of population ageing. [2] Compared to age-matched controls, people with dementia are more likely to require an acute hospital admission,[3 4] more hours of care and longer hospital stays.[5] Hospitalisation also often represents a pivotal event for people with dementia due to increased risk of admission to long-term institutional care, functional decline, mortality, loss of independence, and impact on caregivers.[6] To date most published studies examining hospital admissions of people with dementia have evaluated prevalence samples of people with dementia [7-9]. Other studies have been small, [3 10 11] or have not taken into account the severity of dementia. [3 8 9 12] Some have ascertained admission information from family carers [13 10] which is prone to recall bias.

There is increasing focus on improving interventions and causes of hospitalisation which may be prevented by optimised primary care in the UK for those with dementia.[14] Identifying common causes of hospitalisation in this population could help guide development of preventive strategies to reduce the risk of unnecessary events and/or to avoid lengthy and disruptive admission episodes. However, it is highly likely that the profile of hospitalisations and underlying causal pathways will vary according to the severity of dementia, given the substantial changes that occur during the course of the disease in level of frailty and ability to self-care, not to mention direct effects of neuropsychiatric symptoms on physical health risk. Despite the impact of hospitalisation and the potential for preventative intervention, we are not aware of any previous study that has evaluated the risk and identified the most common causes of

For peer review only - http://bmjopen.bmj.com/site/about/guidelines.xhtml

hospital admission in newly diagnosed dementia and the extent to which this varies with dementia severity.

In this study we sought to evaluate the frequency and common causes of hospitalisation in a newly diagnosed cohort of people with a dementia diagnosis relative to the catchment population, and also the potential association between this relative likelihood of cause-specific hospitalisation and severity of cognitive impairment, using a large cohort drawn from a mental healthcare case register linked to national hospitalisation episodes.

METHODS

Study design and setting

This retrospective observational cohort study involved analysis of newly presenting patients who received a dementia diagnosis. We evaluated hospital episodes in the 12-month period following this index diagnosis date.

The Clinical Record Interactive Search (CRIS) data resource was used to identify dementia cases. CRIS provides research access to anonymised electronic health records (EHR) from the South London and Maudsley NHS Foundation Trust (SLaM). SLaM provides mental healthcare, including dementia assessment and management, for a south London catchment containing approximately 1.2 million residents; EHRs were implemented across all SLaM services from 2006.[15] CRIS data are linked to mortality records and national Hospital Episode Statistics (HES) described below. The

For peer review only - http://bmjopen.bmj.com/site/about/guidelines.xhtml

Oxfordshire Research Ethics Committee C (reference 18/SC/0372) has approved CRIS at the Maudsley as a data resource for secondary analysis.

Routine diagnoses recorded in SLaM are structured according to the WHO International Classification of Diseases 10th edition (ICD-10) and are supplemented in CRIS, by a natural language processing (NLP) algorithm ascertaining diagnoses recorded in correspondence and other text fields.[15 16]. Dementia was defined on the presence of F00*, F01*, F02*or F03* ICD diagnosis codes recorded up to the sixth position in the structured data, or F00,F01, F02, F03 dementia, Alzheimer's disease, Alzheimer's, vascular dementia, mixed dementia from NLP. Hospital outcomes (hospital admissions and its cause) for patients with dementia were obtained from the HES-CRIS linkage. HES contains details of all inpatient admissions at NHS hospitals in England.[17] Discharge diagnoses are recorded as ICD-10 codes and are available for each hospitalisation episode. Additionally, a subset of the HES database detailing hospitalisations for all residents within SLaM's catchment was used in this analysis to generate expected rates for standardisation.

For peer review only

Study participants

We aimed to identify people with newly diagnosed dementia and retrieved records from CRIS for patients with a first recorded diagnosis of dementia between 1 January 2008 and 31 December 2012. We restricted the sample to individuals aged ≥65 years at the time of dementia diagnosis, to those with a measure of cognition within six

For peer review only - http://bmjopen.bmj.com/site/about/guidelines.xhtml

months of their dementia diagnosis, and to those with a Mini Mental State Examination score (MMSE) score of <28 or Health of the Nation Outcome Scales (HoNoS) cognitive impairment score >1. Patients who were receiving care from an acute hospital liaison service at the time of the initial diagnosis were excluded since they reflected cases whose dementia diagnosis might have been precipitated by a hospitalisation and who would have an accompanying higher risk of further events.

Covariates

Age at diagnosis (in five-year bands), sex, ethnicity, and dementia severity (within six months of dementia diagnosis) were extracted from CRIS. Dementia severity was estimated primarily from the MMSE recorded closest to the dementia diagnosis date. If no MMSE score was present within six months of the diagnosis date, the closest-recorded cognitive impairment subscale of HoNOS was used if data on this were available within the six-month period around dementia diagnosis. HoNOS is a clinician-rated instrument usually completed at first assessment, which contains subscales rated 0 (no problem) to 4 (severe or very severe problem), has acceptable/good psychometric properties and correlates with MMSE measurement.[18 19] Dementia severity was categorised as mild, moderate, or severe based on MMSE scores of 21-27, 10-20 and 0-9 which is similar to cut-offs used by NICE, or a HoNOS cognitive impairment subscale score of 2, 3, or 4, respectively.[20]

For peer review only - http://bmjopen.bmj.com/site/about/guidelines.xhtml

All acute general hospital inpatient admissions (both elective and unplanned) for cases were obtained for the 12-month period following the date of dementia diagnosis. Follow-up was censored at the earliest of 31 March 2013 or date of death. Hospitalisations were defined from HES episodes, combining contiguous episodes (i.e. where start and end dates were on the same day). Length of hospital stay (LOS) was defined as the number of days from admission to discharge. The three-digit primary ICD-10 discharge diagnosis was obtained for cases and the catchment population for each hospitalisation. All diagnoses were also grouped at the highest level (letter) ICD-10 code, in line with ICD-10-chapter classifications, broadly related to the body system affected.

Hospitalisations for the catchment population from January 2008 to March 2012 were extracted, and the age and sex profile of the catchment was obtained from the UK Census data for the same time frame. Figure 1 shows the derivation of the case sample.

Statistical analyses

Microsoft Excel and Stata 13.0 were used for analyses. Following a descriptive analysis, we calculated the cumulative incidence of general hospital admissions in the dementia cohort accounting for person-years (py) of follow-up. The association of dementia severity with risk of hospitalisation and length of stay were evaluated by was evaluated by chi squared test and Kruskal Wallis tests respectively. .

For peer review only - http://bmjopen.bmj.com/site/about/guidelines.xhtml
For peer review only

Age- and sex-standardised admission ratios (SARs) with 95% confidence intervals (CI) were calculated overall and for each of the 20 most frequent causes of hospital admission for the 12-month period from the dementia diagnosis based on the three-digit ICD-10 codes. The SAR was defined as the observed number of cases in the dementia cohort admitted with a given cause, divided by the number expected, the second estimate derived from age- and sex-specific rates for admissions with that cause in the SLaM catchment. SARs were also calculated for diagnoses at ICD-10-chapter level. SARs were then calculated separately for each category of dementia severity at the time of dementia diagnosis.

To describe variation in the SAR for each specific cause of admission of interest (three-digit ICD-10 code or ICD-10 chapter) by severity of dementia at diagnosis, linear regression models, with SAR as the dependent variable and dementia stage (mild, moderate, or severe) as the exposure variable, were fitted. The slope of the regression (direction and magnitude) was used to describe the trend across dementia stages for each specific cause as a way in which to quantify whether SARs tended to increase or decrease with dementia severity. In a sensitivity analysis, we excluded repeat admissions (defined as repeat admissions for the same three-digit ICD-10 code in the 12-month period of interest).

For peer review only

For peer review only - http://bmjopen.bmj.com/site/about/guidelines.xhtml

RESULTS

The mean age (SD) of the included patients in the case sample (N=5,218) was 82.2 (7.0) years, and almost two-thirds were women (N=3,338; 64.0%); descriptive characteristics of the analysed cohort by the severity of dementia are displayed in Table 1. At the time of dementia diagnosis, 39.4% were classified as mild, 50.1% as moderate, and 10.4% as severe. The 12-month mortality rate from dementia diagnosis was 15.6% overall: 10.9% for mild severity, 17.5% for moderate, and 24.6% for severe. Approximately half of the patients with dementia were hospitalised during this period (Table 1). While the mean number of admissions did not differ substantially between the dementia severity groups, the median duration of inpatient stay (for all admissions in the 12-month period) increased with higher severity of dementia at diagnosis (p 0.0001). Although the proportion of patients with at least one admission increased with dementia severity, this did not reach statistical significance (p 0.097). Patients with dementia were hospitalised 30% more than the catchment population (SAR: 1.3, 95% CI, 1.2-1.3) during the 12-month follow-up and this SAR was similar across dementia severities: mild 1.2 (95% CI, 1.2-1.2), moderate 1.3 (95% CI, 1.3-1.3), and severe 1.3 (95% CI, 1.2-1.3).

Table 1: Descriptive characteristics of analysis population

	**Dementia Severity**			**All**
	**Mild**	**Moderate**	**Severe**	
	**N=2,057**	**N=2,616**	**N=545**	**N=5,218**

For peer review only

**Mean age (SD)**	81.7 (6.7)	82.6 (7.1)	82.4 (7.5)	82.2 (7.0)
**Sex N (%)**				
Male	765 (37.2)	926 (35.4)	189 (34.7)	1,880 (36.0)
Female	1,292 (62.8)	1,690 (64.6)	356 (65.3)	3,338 (64.0)
**Ethnicity N (%)**				
Afro-Caribbean	219 (10.6)	400 (15.3)	87 (16)	706 (13.5)
Asian	87 (4.2)	105 (4.0)	30 (5.5)	222 (4.3)
European	1,683 (81.8)	2,015 (77.0)	394 (72.3)	4,092 (78.4)
Other	68 (3.3)	96 (3.7)	34 (6.2)	198 (3.8)
**Hospitalisation (12 months after diagnosis)**				
Number of patients with >1 inpatient admission (%)	986 [1](47.9)	1,328 (50.8)	282 (51.7)	2,596 (49.8)
Number of patients per 100 py (95% CI)[1]	54.6 (51.3, 58.1)	60.0 (56.9, 63.3)	63.3 (56.4, 71.2)	58.1 (55.9, 60.4)
**Including readmissions for the same cause**				
Number of hospitalisations[2]	2,413	3,201	664	6,278
Mean number of admissions (SD)	2.4 (5.9)	2.4 (5.9)	2.3 (4.6)	2.4 (5.8)
Median (IQR) inpatient stay	2 (1, 10)	3 (1, 13)	4 (1, 14)	3 (1, 12)
**After excluding readmissions for the same cause**				
Number of hospitalisations [2]	1,868	2,461	508	4,837
Mean number of admissions (SD)	1.9 (1.4)	1.9 (1.3)	1.8 (1.2)	1.9 (1.3)
Median (IQR) inpatient stay	3 (1, 12)	5 (1, 15)	6 (2, 17)	4 (1, 14)

IQR, inter quartile range; py, person years; SD, standard deviation
[1] P value 0.097 (chi squared test)
[2] P value 0.0001 (Kruskal Wallis tests)

The 20 most common three-digit primary discharge diagnoses for hospitalisation episodes in the year following dementia diagnosis are shown in Table 2. The most common post-dementia discharge diagnosis was chronic renal failure, accounting for 27% of the admissions amongst those hospitalised, followed by disorders of the

For peer review only

urinary system (21%). When re-admissions for the same cause were excluded, the most common causes of hospitalisation were disorders of the urinary system, pneumonia, and fracture of the femur, accounting for 15%, 10%, and 6% of admissions, respectively (Table S1). Considering primary discharge diagnoses at ICD-10 chapter level, the highest proportions of admissions were observed in specific diseases of the genitourinary, circulatory and respiratory systems, accounting for 54%, 25%, and 22% of the hospitalisation episodes, respectively (Table 3); however, the broader chapters for 'other symptoms/signs' and 'injury/external cause events' were also common, accounting for 35% and 22% of admissions, respectively. After exclusion of repeat admissions (Table S2), the highest proportions of admissions at a chapter level were in specific diseases of the genitourinary, circulatory and respiratory systems, each accounting for around 20% of the hospitalisation episodes. The broader chapters for 'other symptoms/signs' and 'injury/external cause events', accounted for 31% and 21% of admissions, respectively, after exclusion of repeat admissions at a three-digit ICD level.

For peer review only

Table 2: Standardised admissions ratios by primary discharge diagnosis (20 most common ICD-10 3-digit code) for hospitalisations in the 12 month period following a dementia diagnosis

3-digit ICD primary discharge diagnosis	Number of hospitalisations with this discharge diagnosis	Proportion (%) of episodes with this discharge diagnosis	Standardised admission ratio (95% CI) by dementia severity at diagnosis				Regression coefficient*	P-value*
			All cases	Mild N=986	Moderate N=1,328	Severe N=282		
**Chronic renal failure (N18)**	710	27.3	1.0 (0.9-1.0)	0.8 (0.7-0.9)	1.0 (0.9-1.1)	2.2 (1.9-2.6)	0.71	0.26
**Other disorders of urinary system (N39)**	532	20.5	2.7 (2.5-2.7)	2.4 (2.1-2.8)	2.9 (2.6-3.2)	4.8 (3.9-5.8)	1.18	0.21
**Pneumonia, organism unspecified (J18)**	263	10.1	1.9 (1.7-2.2)	1.4 (1.1-1.8)	2.1 (1.8-2.5)	4.5 (3.5-5.7)	1.56	0.2
**Fracture of femur (S72)**	156	6.0	2.4 (2.0-2.8)	2.4 (1.8-3.1)	2.3 (1.9-2.9)	3.1 (1.9-4.6)	0.32	0.39
**Senility (R54)**	155	6.0	2.9 (2.5-3.4)	2.9 (2.2-3.7)	2.8 (2.2-3.5)	4.6 (3.0-6.7)	0.86	0.36
**Syncope and collapse (R55)**	133	5.1	2.9 (2.4-3.4)	3.0 (2.3-3.9)	2.8 (2.2-3.6)	4.6 (2.9-6.9)	0.77	0.4
**Other cataract (H26)**	132	5.1	0.6 (0.5-0.7)	0.5 (0.4-0.7)	0.6 (0.5-0.8)	0.6 (0.3-1.0)	0.05	0.58
**Unspecified acute lower respiratory infection (J22)**	103	4.0	1.8 (1.5-2.2)	1.4 (1.0-2.0)	1.9 (1.5-2.5)	3.9 (2.5-5.8)	1.24	0.21
**Cerebral infarction (I63)**	100	3.9	1.7 (1.4-2.1)	1.4 (1.0-2.0)	1.8 (1.3-2.3)	3.4 (2.1-5.2)	1.00	0.23
**Other symptoms & signs involving cognitive function and awareness (R41)**	96	3.7	5.2 (4.2-6.3)	4.2 (2.9-6.1)	5.9 (4.5-7.7)	8.1 (4.6-13.2)	1.93	0.05
**Pain in throat and chest (R07)**	91	3.5	1.4 (1.1-1.7)	1.7 (1.2-2.3)	1.3 (1.0-1.8)	1.2 (0.5-2.4)	-0.24	0.16

For peer review only

**Other chronic obstructive pulmonary disease (J44)**	87	3.4	1.0 (0.8-1.2)	1.0 (0.7-1.4)	1.0 (0.8-1.4)	0.8 (0.3-1.6)	-0.13	0.32
**Open wound of head (S01)**	78	3.0	3.0 (2.4-3.8)	3.2 (2.1-4.5)	2.8 (2.0-3.9)	4.5 (2.4-7.7)	0.65	0.47
**Unspecified dementia (F03)**	77	3.0	8.3 (6.6-10.4)	5.0 (2.9-7.9)	10.6 (7.9-14.0)	12.7 (6.8-21.7)	3.87	0.17
**Superficial injury of head (S00)**	73	2.8	3.6 (2.8-4.5)	3.6 (2.4-5.3)	3.3 (2.3-4.5)	6.9 (3.9-11.2)	1.64	0.39
**Heart failure (I50)**	61	2.3	0.8 (0.6-1.1)	0.6 (0.4-1.0)	1.0 (0.7-1.4)	0.8 (0.3-1.7)	0.08	0.76
**Acute renal failure (N17)**	61	2.3	2.1 (1.6-2.8)	1.6 (1.0-2.6)	2.2 (1.5-3.1)	5.7 (3.3-9.2)	2.05	0.25
**Alzheimer's disease (G30)**	60	2.3	10.4 (8.0-13.4)	7.0 (3.9-11.5)	10.8 (7.4-15.2)	31.9 (19.5-49.2)	12.46	0.24
**Other functional intestinal disorders (K59)**	59	2.3	1.8 (1.4-2.3)	2.6 (1.8-3.6)	1.5 (1.0-2.2)	0.9 (0.2-2.5)	-0.85	0.09
**Pneumonitis due to solids and liquids (J69)**	52	2.0	3.0 (2.2-3.9)	1.7 (0.8-3.0)	3.5 (2.4-5.0)	7.9 (4.4-13.1)	3.14	0.15

*The regression coefficient indicates the increase or decrease in standardised admission ratio associated with a one-step increase in severity of dementia. See Statistical analyses section for the linear model relating to these variables. The P-value is for a two-sided test of the null hypothesis regression coefficient = 0.
CI, confidence interval

For peer review only

Table 3: Standardised admissions ratios (95% CI) by discharge diagnosis (ICD-10 chapter) for hospitalisations in the 12 month period after a dementia diagnosis

Discharge diagnosis by ICD-10 chapter		Number of hospitalisations with this discharge diagnosis	Proportion (%) of episodes with this discharge diagnosis	Standardised admission ratio (95% CI) by dementia severity at diagnosis				Regression Coefficient*	P>|t|*
				All cases	Mild N=986	Moderate N=1,328	Severe N=282		
**A**	Infectious and parasitic diseases	62	2.4	1.5 (1.2-2.0)	1.0 (0.6-1.7)	1.8 (1.3-2.5)	2.1 (0.9-3.9)	0.51	0.16
**B**	Infectious and parasitic diseases	11	0.4	1.6 (0.8-2.9)	0.8 (0.1-2.8)	2.1 (0.8-4.2)	2.8 (0.3-10.0)	0.99	0.11
**C**	Cancers	167	6.4	0.3 (0.3-0.4)	0.3 (0.3-0.4)	0.4 (0.3-0.5)	0.1 (0.1-0.3)	-0.1	0.47
**D**	Benign neoplasms or diseases of the blood	158	6.1	0.8 (0.7-1.0)	0.9 (0.7-1.1)	0.9 (0.7-1.1)	0.3 (0.1-0.7)	-0.3	0.31
**E**	Endocrine, nutritional and metabolic diseases	138	5.3	1.9 (1.6-2.3)	1.9 (1.5-2.5)	1.9 (1.4-2.4)	2.1 (1.2-3.5)	0.09	0.54
**F**	Mental and behavioural disorders	194	7.5	5.7 (4.9-6.6)	4.7 (3.6-6.1)	6.1 (5-7.4)	7.4 (4.9-10.8)	1.35	0.01
**G**	Diseases of the nervous system	183	7.0	2.4 (2.1-2.8)	2.1 (1.6-2.7)	2.4 (2.0-3.0)	3.4 (2.3-5.0)	0.65	0.18
**H**	Diseases of the eye	209	8.1	0.6 (0.5-0.7)	0.6 (0.5-0.8)	0.7 (0.5-0.8)	0.3 (0.2-0.6)	-0.13	0.43
**I**	Diseases of the circulatory system	638	24.6	1.3 (1.2-1.3)	1.4 (1.2-1.5)	1.2 (1.1-1.4)	1.0 (0.8-1.4)	-0.16	0.06
**J**	Diseases of the respiratory system	567	21.8	1.6 (1.5-1.6)	1.3 (1.1-1.5)	1.7 (1.5-1.9)	2.2 (1.8-2.7)	0.46	0.03
**K**	Diseases of the digestive system	448	17.3	1.0 (0.9-1.0)	1.2 (1.1-1.4)	1.0 (0.8-1.1)	0.6 (0.4-0.9)	-0.3	0.06
**L**	Diseases of the skin	105	4.0	1.2 (1.0-1.5)	1.2 (0.9-1.6)	1.1 (0.8-1.4)	1.9 (1.1-3.0)	0.33	0.41

For peer review only

**M**	Diseases of the musculoskeletal system	239	9.2	0.9 (0.8-1.1)	1.1 (0.9-1.3)	0.8 (0.7-1.0)	0.9 (0.6-1.4)	-0.07	0.64
**N**	Diseases of the genitourinary system	1389	53.5	1.3 (1.2-1.3)	1.1 (1.0-1.1)	1.4 (1.3-1.4)	1.7 (1.4-1.9)	0.28	0.01
**R**	Symptoms and sign not elsewhere classified	913	35.2	1.9 (1.8-1.9)	2 (1.8-2.2)	1.9 (1.7-1.9)	1.7 (1.3-2.1)	-0.15	0.18
**S**	Injury, poisoning and certain other external causes	574	22.1	2.3 (2.1-2.3)	2.3 (2.0-2.7)	2.2 (2.0-2.5)	2.4 (1.9-3.1)	0.05	0.69
**T**	Injury, poisoning and certain other external causes	120	4.6	1.6 (1.3-1.9)	1.6 (1.2-2.1)	1.8 (1.4-2.3)	0.7 (0.2-1.5)	-0.46	0.47
**Z**	External causes	161	6.2	1.1 (1.0-1.3)	1.6 (1.3-1.9)	0.9 (0.7-1.1)	0.8 (0.4-1.4)	-0.4	0.25
**Total**		2,596	-	1.3 (1.2-1.3)	1.2 (1.2-1.2)	1.3 (1.3-1.3)	1.3 (1.2-1.3)	0.03	0.3

*The regression coefficient indicates the increase or decrease in SAR associated with a one-step increase in severity of dementia. See Statistical analyses section for the linear model relating to these variables. The P-value is for a two-sided test of the null hypothesis regression coefficient = 0.

CI, confidence interval

For peer review only

SARs for each discharge diagnosis by the severity of dementia at diagnosis, are also shown in Tables 2 and 3, and are illustrated in Figures 2 and 3. For the cohort as a whole, patients with dementia were hospitalised more than the catchment population (SAR significantly greater than 1) for most of the 20 most common discharge diagnoses, the exceptions being chronic renal failure (N18), other chronic obstructive pulmonary disease (J44), heart failure (I50) (SARs not significantly >1), and other cataracts (H26, SAR <1). The trends across dementia severity groups were positive for 17 of the 20 conditions (increased SARs with increasing severity, indicated by a positive regression coefficient); however, only the trend for the R41 code ("other symptoms and signs involving cognitive function and awareness") was statistically significant. Considering ICD chapters, in most groups, patients with dementia were hospitalised more than the catchment population (Table 3). The exceptions were cancers, benign neoplasms and diseases of the eye (SAR < 1). Of the 18 ICD-10 chapters, trends across dementia severity groups were positive in 10; however, only those for mental and behavioural disorders (F), diseases of the respiratory system (J), and diseases of the genitourinary system (N) were statistically significant. No chapters showed significantly decreasing SARs with increasing dementia severity, although the negative trend for diseases of the digestive system (K) was close to significance. After excluding repeat admissions for the same cause, similar trends were observed at the 3-digit ICD and chapter level, although an increasing trend of SAR with dementia severity reached or approached statistical significance for disorders of the urinary system and pneumonia with an increasing trend with dementia severity, and a decreasing trend, which reached statistical significance, for pain in throat and chest (Tables S3-4, Figures 2-3).

For peer review only

For peer review only - http://bmjopen.bmj.com/site/about/guidelines.xhtml

For peer review only

For peer review only - http://bmjopen.bmj.com/site/about/guidelines.xhtml

DISCUSSION

In this retrospective cohort study of people with a dementia diagnosis, over half had at least one hospitalisation in the 12 months after diagnosis. The risk of hospitalisation and the length of hospital stay increased with higher dementia severity, although risk of hospitalisation did not reach statistical significance. Most of the common reasons for hospitalisation, based on the primary discharge diagnosis, were also more common than expected in cases relative to the catchment population. Differences in SARs by baseline dementia severity, where present, were more evident for discharge diagnoses grouped at ICD-10-chapter level than at the level of specific 3-digit ICD-10 codes.

The hospitalisation rate for at least one inpatient admission in the 12-month period after dementia diagnosis was 58 per 100 py, slightly higher than reported elsewhere..[21 22] This may reflect more recent data (given that hospitalisation rates are increasing), the near-complete data on hospitalisation in our study, and/or the fact that hospitalisation is free at the point of delivery in the UK context.[7] Increasing likelihood and length of hospital stay with increasing dementia severity is consistent with other reports, although differences between admissions from community and institutional facilities have been described, with reduced healthcare use following relocation to institutional care, potentially reflecting higher support.[23-25] This, as well as survival effects, may account for our finding that the most marked difference in hospitalisation was between mild and moderate dementia rather than between moderate and severe dementia.

The most common causes of hospital admission, after excluding repeat admissions for the same cause, were disorders of the urinary system and pneumonia, followed by fracture of femur, senility and syncope and collapse; this is similar to findings reported

For peer review only - http://bmjopen.bmj.com/site/about/guidelines.xhtml

elsewhere.[4 9 26-28] Most of the common causes of admission identified in people with dementia were more frequent than expected relative to the catchment population (SAR > 1).

Higher risk of syncope and collapse, and of other diagnoses related to fractures and falls, have been previously reported in patients with dementia,[4 9 26] and may reflect age-related autonomic dysfunction, comorbid disorders and polypharmacy,[29] impaired visuospatial functions and gait instability.[30] A review of published clinical trials of atypical anti-psychotic medication use did not find evidence of increased injury, falls, or syncope associated with their use,[31] nor did a recent study of hospitalised falls/fractures in a CRIS-derived cohort of people with dementia.[32] Significant predictors included social/demographic factors, physical health problems, and previous episode; no associations were found with neuropsychiatric symptoms, cognitive (MMSE) scores, or functional problems. [32] Inpatient admissions due to infection were also more common than expected, both at the level of specific disorders and at the ICD-10-chapter level. This may reflect more rapid age-related decline in immune function,[33] problems with mobility associated with urinary incontinence and subsequent infection,[34] or inactivity and dysphagia causing respiratory tract infection.[35] Acute renal failure is reported to be associated with a high risk of hospital re-admission.[36 37] In our study it remained a common cause of admission amongst individuals with dementia even after excluding repeat admissions. Risk factors for acute renal failure include hospitalisation, infection, diabetes, pre-existing renal impairment and advanced age.[38] which themselves may be associated with dementia severity.

A lower than expected frequency of hospitalisation (SAR <1) was observed for heart failure, cancers, benign neoplasms and diseases of the eye, consistent with other reports,[21 28] and possibly reflecting diagnostic delay. For example, an association of dementia with later-stage cancer at diagnosis has been reported,[39] as well as unexpected cases being identified at autopsy;[40] furthermore, patients with dementia

For peer review only - http://bmjopen.bmj.com/site/about/guidelines.xhtml

may be less likely to undergo invasive diagnostic tests and receive fewer treatment interventions, potentially explaining a shorter duration of survival.[41]

While rates of healthcare utilisation and costs, or limited specified causes of admission,[23 24] have been reported in relation to severity of dementia, we are not aware of any evaluation of common causes of hospital admission by dementia stage. Most of the SARs for specific causes of hospitalisation were positively associated with dementia severity, although most not to a significant extent. Associations, where significant, were more often with discharge diagnoses categorised by ICD-10 chapter rather than 3-character code. This may have arisen because of higher statistical power for the chapter-level comparisons but might also reflect differences in the accuracy and/or variability of code assignments in routine administrative data; for example, there may be differences between hospitals in which specific 3-character code to assign to a particular hospitalisation profile but stronger agreement on the broader chapter heading.

As well as hospitalisations as a direct result of dementia, with worsening cognitive impairment, there may be an increase in admissions to acute care for exacerbation, due to an individual's decreasing ability to manage existing comorbidity. Similarly, if identification of new comorbidity is delayed at earlier stages of dementia, this may increase the risk of hospitalisations later in its course due to under-recognised and/or under-treated disorders, although there is also evidence for an increased risk of some causes of hospitalisation even prior to a dementia diagnosis.[42] An increased length of stay is consistent with the higher rate of complications seen in hospitalised patients with dementia, as well as with increasing severity of dementia.[43] Increasing respiratory and genitourinary disease admissions most likely represent infections, and diseases of the nervous system may reflect cases where the dementia itself has been provided as a primary diagnosis, or else comorbidities such as depression, psychosis or other behavioural/psychological manifestations. Risk for diseases of the digestive system

For peer review only

For peer review only - http://bmjopen.bmj.com/site/about/guidelines.xhtml

diminished across the severity groups, but this may reflect a diminished ability to communicate non-specific symptoms rather than a reduced risk of defined disorders, as indicated by the negative coefficient for 'other functional intestinal disorders' (K59) in Table 2, which was the most common digestive system diagnosis.

Strengths of the study included the large and representative sample of diagnosed cases of dementia and the linkage to a national hospitalisation database providing near-complete outcome ascertainment. Patients who were receiving care from acute hospital liaison services at the time of their dementia diagnosis were excluded from the study since these may reflect individuals whose diagnosis might have been precipitated by the hospitalisation, and yet who would also have an increased risk of re-hospitalisation by virtue of their status, thus biasing the association of interest. Considering limitations, the analysed sample was from a single service and also only included cases with dementia diagnosed in specialist services; however, estimated proportions of people with dementia in the SLaM catchment who receive a specialist diagnosis is relatively high at 75.2%, compared to 67.6% nationally.[44] This study was descriptive in nature and used all available data; as such it was not generated with a specific power calculation for the associations of interest, and the relatively small number of cases with severe dementia at diagnosis will have limited the detection of differences across the three groups. Furthermore, unrecognised dementia cases omitted from observed admission rates will have biased findings towards the null, as would healthy survivor effects (which may also be particularly an issue for the severe dementia group). We used the discharge diagnosis recorded during the last episode of the hospital spell, and it is possible that the primary discharge diagnosis code may reflect complications that arose during the hospital stay rather than the reason for initial hospital admission. Although we compared SARs across dementia severities for a given discharge diagnosis, due to potential differences in the underlying age structure between severity categories SARS are not strictly comparable with each other. However, as the mean age was broadly similar between dementia severities,

For peer review only - http://bmjopen.bmj.com/site/about/guidelines.xhtml

this mitigates this concern in part. Finally, this analysis did not attempt to account for comorbidity, medication use, institutional residence or socioeconomic status, did not sub-classify dementia, and was not able to investigate or take into account clustering by admission units.

Understanding the factors influencing health service utilisation by patients with dementia is necessary to inform care needs and to guide future healthcare resource planning and allocation. Our findings are of importance given that hospitalisation is a significant element in the cost of dementia care and that the prevalence of dementia is increasing. Our study highlights the need to develop specific strategies for those causes of hospitalisation that are most amenable to prevention in dementia. Further research on factors influencing patterns of healthcare use over time and severity would be useful in this context.

Figure 1: Identification of the case sample

Figure 2: Standardised admissions ratios by 3-digit ICD code for mild, moderate and severe dementia populations (excludes repeat admissions for the same cause at a 3-digit ICD level)

Figure 3: Standardised admissions ratios by ICD chapter for mild, moderate and severe dementia populations (excludes repeat admissions for the same cause at a 3-digit ICD level)

Patient and Public Involvement

For peer review only - http://bmjopen.bmj.com/site/about/guidelines.xhtml

No patient involved

Funding

This work was supported by NIHR Biomedical Research Centre at the South London and Maudsley NHS Foundation Trust and King's College London. RS is additionally part-funded by a Medical Research Council Mental Health Data Pathfinder Award to King's College London, and an NIHR Senior Investigator Award

Role of the funding source

The funder of the study had no role in the study design, data collection, data analysis, data interpretation, or writing of the report. UG, GP and RS had access to the anonymised data. The corresponding author had the final responsibility for the decision to submit for publication.

Declaration of Competing Interest

UG and NG are employees of GSK, hold stock and receive a salary from GSK. RS has received research funding in the last 5 years from Roche, Janssen, Takeda and GSK.

Contributorship statement

UG was responsible for the design, analysis, interpretation and drafting of the manuscript. GP contributed to the design and analysis of the study. NG provided

For peer review only

statistical input and contributed to the drafting of the manuscript. RS contributed to the design, interpretation and drafting of the manuscript

Acknowledgments

The authors would like to acknowledge Hitesh Shetty for his contributions to the study.

Data sharing

Because of their nature and to comply with their ethical approval, CRIS data are required to remain within the firewall of the South London and Maudsley NHS Foundation Trust (SLaM). Access to the data used for this study can be facilitated by the CRIS Oversight Committee on application and with appropriate SLaM affiliation, details of which can be obtained from cris.administrator@slam.nhs.uk.

For peer review only

References:

1. Alzheimer's_Society. Dementia UK Update, 2014.
2. Ahmadi-Abhari S, Guzman-Castillo M, Bandosz P, et al. Temporal trend in dementia incidence since 2002 and projections for prevalence in England and Wales to 2040: modelling study. *BMJ* 2017;358:j2856. doi: 10.1136/bmj.j2856 [published Online First: 2017/07/07]
3. Phelan EA, Borson S, Grothaus L, et al. Association of incident dementia with hospitalizations. *JAMA - Journal of the American Medical Association* 2012;307(2):165-72. doi: 10.1001/jama.2011.1964

For peer review only - http://bmjopen.bmj.com/site/about/guidelines.xhtml

4. Natalwala A, Potluri R, Uppal H, et al. Reasons for hospital admissions in dementia patients in Birmingham, UK, during 2002-2007. *Dementia and Geriatric Cognitive Disorders* 2008;26(6):499-505. doi: 10.1159/000171044
5. Mukadam N, Sampson EL. A systematic review of the prevalence, associations and outcomes of dementia in older general hospital inpatients. *Int Psychogeriatr* 2011;23(3):344-55. doi: 10.1017/S1041610210001717 [published Online First: 2010/08/19]
6. Lehmann J, Michalowsky B, Kaczynski A, et al. The impact of hospitalization on readmission, institutionalization, and mortality of people with dementia: A systematic review and meta-analysis. *Journal of Alzheimer's Disease* 2018;64(3):735-49. doi: 10.3233/JAD-171128
7. Bernardes C, Massano J, Freitas A. Hospital admissions 2000–2014: A retrospective analysis of 288 096 events in patients with dementia. *Archives of Gerontology and Geriatrics* 2018;77:150-57. doi: 10.1016/j.archger.2018.05.006
8. Tolppanen AM, Taipale H, Purmonen T, et al. Hospital admissions, outpatient visits and healthcare costs of community-dwellers with Alzheimer's disease. *Alzheimer's and Dementia* 2015;11(8):955-63. doi: 10.1016/j.jalz.2014.10.005
9. Draper B, Karmel R, Gibson D, et al. The Hospital Dementia Services Project: age differences in hospital stays for older people with and without dementia. *Int Psychogeriatr* 2011;23(10):1649-58. doi: 10.1017/S1041610211001694 [published Online First: 2011/09/10]
10. Voisin T, Andrieu S, Cantet C, et al. Predictive factors of hospitalizations in Alzheimer's disease: A two-year prospective study in 686 patients of the REAL.FR study. *Journal of Nutrition, Health and Aging* 2010;14(4):288-91. doi: 10.1007/s12603-010-0063-4
11. Russ TC, Parra MA, Lim AE, et al. Prediction of general hospital admission in people with dementia: cohort study. *Br J Psychiatry* 2015;206(2):153-9. doi: 10.1192/bjp.bp.113.137166 [published Online First: 2014/11/15]
12. Joyce AT, Zhao Y, Bowman L, et al. Burden of illness among commercially insured patients with Alzheimer's disease. *Alzheimers Dement* 2007;3(3):204-10. doi: 10.1016/j.jalz.2007.04.373
13. Andrieu S, Reynish E, Nourhashemi F, et al. Predictive factors of acute hospitalization in 134 patients with Alzheimer's disease: a one year prospective study. *Int J Geriatr Psychiatry* 2002;17(5):422-6. doi: 10.1002/gps.624 [published Online First: 2002/05/08]
14. Department_of_Health. Living well with dementia: A national dementia strategy: Department of Health 2009., 2009.
15. Perera G, Broadbent M, Callard F, et al. Cohort profile of the South London and Maudsley NHS Foundation Trust Biomedical Research Centre (SLaM BRC) Case Register: current status and recent enhancement of an Electronic Mental Health Record-derived data resource. *BMJ Open* 2016;6(3):e008721. doi: 10.1136/bmjopen-2015-008721 [published Online First: 2016/03/05]
16. Jackson RG, Patel R, Jayatilleke N, et al. Natural language processing to extract symptoms of severe mental illness from clinical text: the Clinical Record Interactive Search Comprehensive Data Extraction (CRIS-CODE) project. *BMJ Open* 2017;7(1):e012012. doi: 10.1136/bmjopen-2016-012012 [published Online First: 2017/01/18]

For peer review only

17. NHS. Hospital Episode Statistics (HES) 2019 [Available from: https://digital.nhs.uk/data-and-information/data-tools-and-services/data-services/hospital-episode-statistics accessed 13/01/2019 2019.
18. Pirkis JE, Burgess PM, Kirk PK, et al. A review of the psychometric properties of the Health of the Nation Outcome Scales (HoNOS) family of measures. *Health Qual Life Outcomes* 2005;3:76. doi: 10.1186/1477-7525-3-76 [published Online First: 2005/11/30]
19. Canuto A, Weber K, Gold G, et al. Structured assessment of mental health status in psychogeriatrics: validity of the French HoNOS65+. *Can J Psychiatry* 2007;52(1):37-45. doi: 10.1177/070674370705200107 [published Online First: 2007/04/21]
20. NICE. Donepezil, galantamine, rivastigmine and memantine for the treatment of dementia. Health technology appraisal 2018 update. In: NICE, ed. 2018 ed, 2018.
21. Tuppin P, Kusnik-Joinville O, Weill A, et al. Primary health care use and reasons for hospital admissions in dementia patients in france: database study for 2007. *Dement Geriatr Cogn Disord* 2009;28(3):225-32. doi: 10.1159/000238394 [published Online First: 2009/09/25]
22. Chen L, Reed C, Happich M, et al. Health care resource utilisation in primary care prior to and after a diagnosis of Alzheimer's disease: a retrospective, matched case-control study in the United Kingdom. *BMC geriatrics* 2014;14:76. doi: 10.1186/1471-2318-14-76
23. Lin PJ, Rane PB, Fillit HM, et al. National estimates of potentially avoidable hospitalizations among medicare beneficiaries with Alzheimer's disease and related dementias. *Alzheimer's and Dementia* 2016;12(7):P253-P54.
24. Zhu CW, Cosentino S, Ornstein K, et al. Use and cost of hospitalization in dementia: Longitudinal results from a community-based study. *International Journal of Geriatric Psychiatry* 2015;30(8):833-41. doi: 10.1002/gps.4222
25. Fillenbaum G, Heyman A, Peterson BL, et al. Use and cost of hospitalization of patients with AD by stage and living arrangement: CERAD XXI. *Neurology* 2001;56(2):201-6. [published Online First: 2001/02/13]
26. Zhao Y, Kuo TC, Weir S, et al. Healthcare costs and utilization for Medicare beneficiaries with Alzheimer's. *BMC Health Services Research* 2008;8 doi: 10.1186/1472-6963-8-108
27. Rao A, Suliman A, Vuik S, et al. Outcomes of dementia: Systematic review and meta-analysis of hospital administrative database studies. *Arch Gerontol Geriatr* 2016;66:198-204. doi: 10.1016/j.archger.2016.06.008
28. Guijarro R, San Roman CM, Gomez-Huelgas R, et al. Impact of dementia on hospitalization. *Neuroepidemiology* 2010;35(2):101-8. doi: 10.1159/000311032 [published Online First: 2010/06/17]
29. O' Brien H, Kenny A. Syncope in the Elderly. *Eur Cardiol* 2014;9(1):28-36. doi: 10.15420/ecr.2014.9.1.28 [published Online First: 2014/07/01]
30. Sheridan PL, Hausdorff JM. The role of higher-level cognitive function in gait: executive dysfunction contributes to fall risk in Alzheimer's disease. *Dement Geriatr Cogn Disord* 2007;24(2):125-37. doi: 10.1159/000105126
31. Schneider LS, Dagerman K, Insel PS. Efficacy and adverse effects of atypical antipsychotics for dementia: meta-analysis of randomized, placebo-controlled trials. *Am J Geriatr Psychiatry* 2006;14(3):191-210. doi: 10.1097/01.JGP.0000200589.01396.6d [published Online First: 2006/03/01]

For peer review only - http://bmjopen.bmj.com/site/about/guidelines.xhtml

32. Sharma S, Mueller C, Stewart R, et al. Predictors of Falls and Fractures Leading to Hospitalization in People With Dementia: A Representative Cohort Study. *Journal of the American Medical Directors Association* 2018;19(7):607-12. doi: 10.1016/j.jamda.2018.03.009
33. Janssens JP. Pneumonia in the elderly (geriatric) population. *Curr Opin Pulm Med* 2005;11(3):226-30. [published Online First: 2005/04/09]
34. Schnelle JF, Leung FW. Urinary and fecal incontinence in nursing homes. *Gastroenterology* 2004;126(1 Suppl 1):S41-7. [published Online First: 2004/02/24]
35. Palmer JL, Metheny NA. Preventing aspiration in older adults with dysphagia. *Am J Nurs* 2008;108(2):40-8; quiz 49. doi: 10.1097/01.NAJ.0000308961.99857.33 [published Online First: 2008/01/30]
36. Jencks SF, Williams MV, Coleman EA. Rehospitalizations among patients in the Medicare fee-for-service program. *N Engl J Med* 2009;360(14):1418-28. doi: 10.1056/NEJMsa0803563 [published Online First: 2009/04/03]
37. Silver SA, Harel Z, McArthur E, et al. 30-Day Readmissions After an Acute Kidney Injury Hospitalization. *Am J Med* 2017;130(2):163-72 e4. doi: 10.1016/j.amjmed.2016.09.016 [published Online First: 2016/10/19]
38. Hilton R. Acute renal failure. *BMJ* 2006;333(7572):786-90. doi: 10.1136/bmj.38975.657639.AE [published Online First: 2006/10/14]
39. Gorin SS, Heck JE, Albert S, et al. Treatment for breast cancer in patients with Alzheimer's disease. *J Am Geriatr Soc* 2005;53(11):1897-904. doi: 10.1111/j.1532-5415.2005.00467.x [published Online First: 2005/11/09]
40. Gupta SK, Lamont EB. Patterns of presentation, diagnosis, and treatment in older patients with colon cancer and comorbid dementia. *J Am Geriatr Soc* 2004;52(10):1681-7. doi: 10.1111/j.1532-5415.2004.52461.x [published Online First: 2004/09/29]
41. Libert Y, Dubruille S, Borghgraef C, et al. Vulnerabilities in Older Patients when Cancer Treatment is Initiated: Does a Cognitive Impairment Impact the Two-Year Survival? *PLoS One* 2016;11(8):e0159734. doi: 10.1371/journal.pone.0159734 [published Online First: 2016/08/02]
42. Desai U, Kirson NY, Mehta N, et al. Trends in health service use and potentially avoidable hospitalizations before Alzheimer's disease diagnosis: A matched, retrospective study of US Medicare beneficiaries. *Alzheimer's & Dementia Diagnosis, Assessment & Disease Monitoring* 2018;11(1)
43. Möllers T, Stocker H, Wei W, et al. Length of hospital stay and dementia: A systematic review of observational studies. *International Journal of Geriatric Psychiatry* 2019;34(1):8-21. doi: 10.1002/gps.4993
44. NHS_England. Dementia diagnosis monthly workbook: Secondary dementia diagnosis monthly workbook 2017, 2017.

For peer review only

For peer review only - http://bmjopen.bmj.com/site/about/guidelines.xhtml

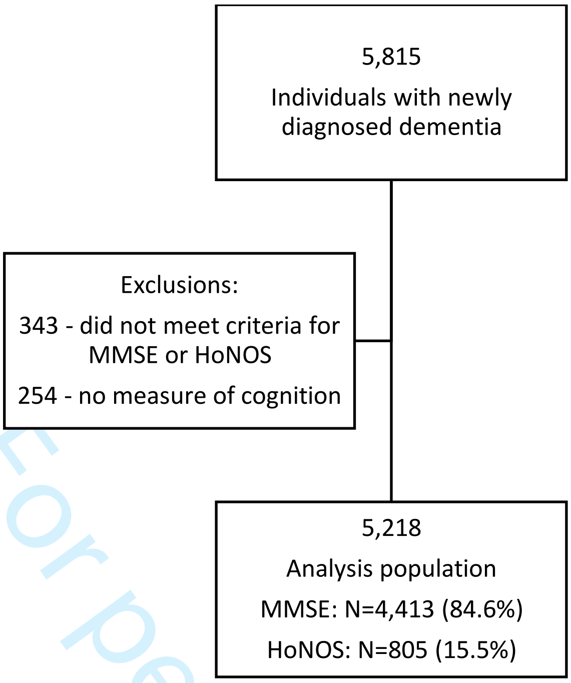

For peer review only

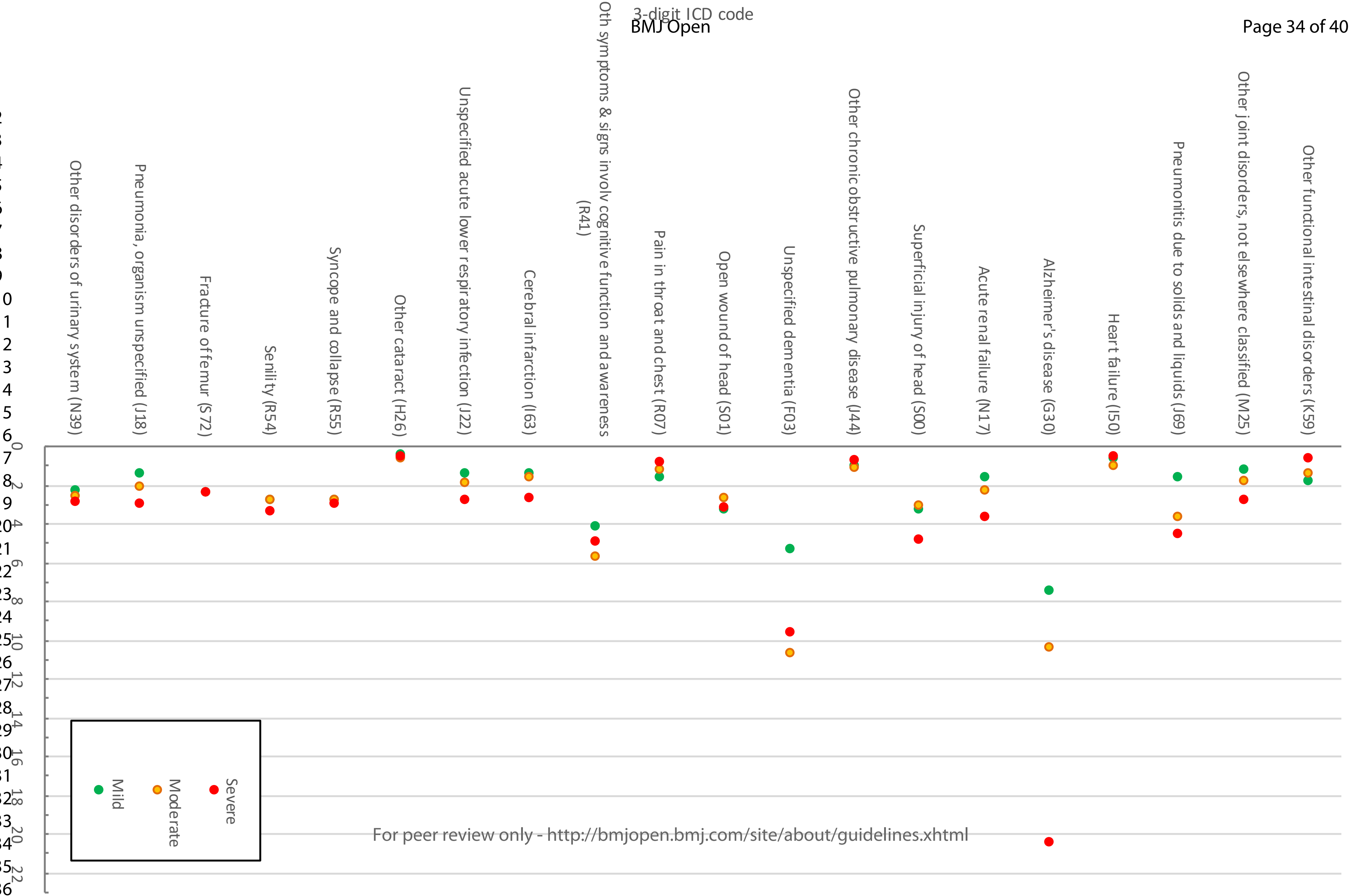

For peer review only - http://bmjopen.bmj.com/site/about/guidelines.xhtml

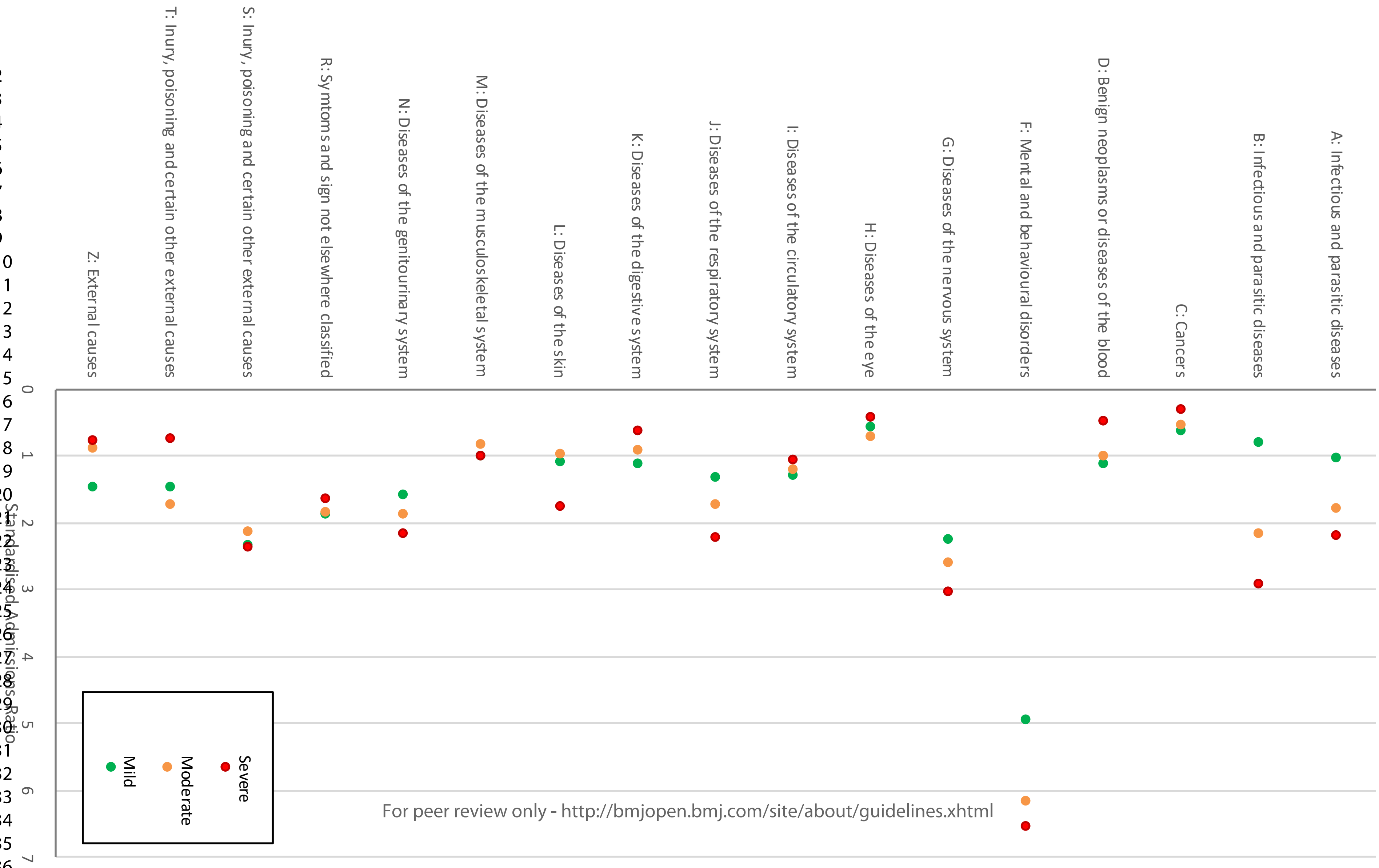
Chapter
Z: External causes
T: Inury, poisoning and certain other external causes
S: Inury, poisoning and certain other external causes
R: Symtoms and sign not elsewhere classified
N: Diseases of the genitourinary system
M: Diseases of the musculoskeletal system
L: Diseases of the skin
K: Diseases of the digestive system
J: Diseases of the respiratory system
I: Diseases of the circulatory system
H: Diseases of the eye
G: Diseases of the nervous system
F: Mental and behavioural disorders
D: Benign neoplasms or diseases of the blood
C: Cancers
B: Infectious and parasitic diseases
A: Infectious and parasitic diseases
Standardised Admissions Ratio
0
1
2
3
4
5
6
7
Mild
Moderate
Severe

Table S1: **Twenty most common primary discharge diagnoses amongst individuals with dementia receiving inpatient care in the 12 months after dementia diagnosis (repeat admissions excluded)**

Primary discharge diagnosis (ICD code)*	Number of episodes	% of total inpatient care episodes
Other disorders of urinary system (N39)	399	15.4
Pneumonia, organism unspecified (J18)	248	9.6
Fracture of femur (S72)	150	5.8
Senility (R54)	128	4.9
Syncope and collapse (R55)	117	4.5
Other cataract (H26)	107	4.1
Unspecified acute lower respiratory infection (J22)	97	3.7
Cerebral infarction (I63)	86	3.3
Other symptoms & signs involving cognitive function and awareness (R41)	86	3.3
Pain in throat and chest (R07)	78	3.0
Open wound of head (S01)	75	2.9
Unspecified dementia (F03)	73	2.8
Other chronic obstructive pulmonary disease (J44)	71	2.7
Superficial injury of head (S00)	67	2.6
Acute renal failure (N17)	57	2.2
Alzheimer's disease (G30)	56	2.2
Heart failure (I50)	52	2.0
Pneumonitis due to solids and liquids (J69)	49	1.9
Other joint disorders, not elsewhere classified (M25)	46	1.8
Other functional intestinal disorders (K59)	43	1.7

*Three digit ICD-10 discharge diagnoses; repeat admissions for the same diagnosis removed.
Percentages based on 2,596 individuals with at least 1 inpatient admission in the 12 month period after the dementia diagnosis date

For peer review only

Table S2: **ICD chapter level primary discharge diagnoses amongst individuals with dementia receiving inpatient care in the 12 months after dementia diagnosis (repeat episodes excluded)***

**Chapter**		**Number of episodes**	**% of total inpatient care episodes**
**A**	Infectious and parasitic diseases	59	2.3
**B**	Infectious and parasitic diseases	11	0.4
**C**	Cancers	123	4.7
**D**	Benign neoplasms or diseases of the blood	119	4.6
**E**	Endocrine, nutritional and metabolic diseases	122	4.7
**F**	Mental and behavioural disorders	180	6.9
**G**	Diseases of the nervous system	159	6.1
**H**	Diseases of the eye	166	6.4
**I**	Diseases of the circulatory system	542	20.9
**J**	Diseases of the respiratory system	514	19.8
**K**	Diseases of the digestive system	395	15.2
**L**	Diseases of the skin	89	3.4
**M**	Diseases of the musculoskeletal system	214	8.2
**N**	Diseases of the genitourinary system	554	21.3
**R**	Symptoms and signs not elsewhere classified	803	30.9
**S**	Injury, poisoning and certain other external causes	545	21.0
**T**	Injury, poisoning and certain other external causes	100	3.9
**Z**	External causes	140	5.4

*Percentages based on 2,596 individuals with at least 1 inpatient admission in the 12 month period after the dementia diagnosis date. Repeat episodes for the same 3-digit ICD code in the 12-month time period excluded

For peer review only

Table S3: Standardised admissions ratios (95% CI) for hospitalisations in the 12 months following a first dementia diagnosis – by 3 digit ICD-10 code for the primary discharge diagnosis* (repeat admissions excluded)

Primary discharge diagnosis	By dementia severity at first diagnosis					
	All	Mild	Moderate	Severe	Coefficient†	P-value†
Other disorders of urinary system (N39)	2.5 (2.3-2.5)	2.3 (2.0-2.7)	2.6 (2.3-3.0)	2.9 (2.2-3.8)	0.29	**0.01**
Pneumonia, organism unspecified (J18)	2.0 (1.7-2.3)	1.5 (1.2-1.9)	2.1 (1.8-2.5)	3.0 (2.2-4.1)	0.77	0.07
Fracture of femur (S72)	2.4 (2.0-2.8)	2.4 (1.8-3.2)	2.4 (1.9-3.0)	2.5 (1.4-3.9)	0.01	0.8
Senility (R54)	2.8 (2.4-3.4)	2.8 (2.0-3.7)	2.8 (2.1-3.5)	3.4 (2.0-5.5)	0.32	0.33
Syncope and collapse (R55)	2.8 (2.3-3.4)	2.8 (2.1-3.8)	2.8 (2.1-3.6)	3.0 (1.6-5.1)	0.07	0.44
Other cataract (H26)	0.6 (0.5-0.7)	0.5 (0.3-0.7)	0.7 (0.5-0.9)	0.5 (0.3-1.0)	0.04	0.8
Unspecified acute lower respiratory infection (J22)	1.9 (1.5-2.3)	1.5 (1.0-2.1)	1.9 (1.4-2.5)	2.8 (1.6-4.6)	0.66	0.12
Cerebral infarction (I63)	1.7 (1.3-2.1)	1.4 (0.9-2.1)	1.6 (1.2-2.2)	2.7 (1.5-4.5)	0.65	0.24
Other symptoms & signs involving cognitive function and awareness (R41)	5.1 (4.0-6.2)	4.2 (2.7-6.1)	5.7 (4.3-7.6)	4.9 (2.3-9.4)	0.39	0.67
Pain in throat and chest (R07)	1.4 (1.1-1.7)	1.7 (1.2-2.3)	1.2 (0.9-1.7)	0.8 (0.3-2.0)	-0.42	**0.03**
Open wound of head (S01)	3.0 (2.4-3.7)	3.3 (2.2-4.7)	2.7 (1.9-3.8)	3.2 (1.5-6.1)	-0.04	0.91
Unspecified dementia (F03)	8.6 (6.7-10.8)	5.4 (3.1-8.6)	10.7 (7.9-14.2)	9.6 (4.4-18.3)	2.11	0.46
Other chronic obstructive pulmonary disease (J44)	1.1 (0.8-1.4)	1.1 (0.7-1.6)	1.1 (0.8-1.5)	0.7 (0.2-1.7)	-0.18	0.35
Superficial injury of head (S00)	3.4 (2.6-4.3)	3.3 (2.1-5.0)	3.1 (2.1-4.4)	4.9 (2.5-8.8)	0.79	0.41
Acute renal failure (N17)	2.2 (1.7-2.9)	1.6 (0.9-2.6)	2.4 (1.6-3.3)	3.7 (1.8-6.8)	1.05	0.1
Alzheimer's disease (G30)	10.4 (7.9-13.6)	7.5 (4.2-12.3)	10.5 (7.0-15.0)	20.5 (10.6-35.8)	6.5	0.19
Heart failure (I50)	0.9 (0.6-1.1)	0.6 (0.4-1.1)	1.1 (0.7-1.5)	0.6 (0.2-1.6)	-0.02	0.96
Pneumonitis due to solids and liquids (J69)	3.0 (2.2-4.0)	1.6 (0.8-3.0)	3.7 (2.5-5.2)	4.6 (2.0-9.0)	1.46	0.14
Other joint disorders, not elsewhere classified (M25)	1.7 (1.3-2.3)	1.3 (0.7-2.2)	1.8 (1.2-2.7)	2.8 (1.2-5.6)	0.79	0.09
Other functional intestinal disorders (K59)	1.5 (1.1-2.0)	1.8 (1.1-2.8)	1.4 (0.9-2.2)	0.7 (0.1-2.4)	-0.58	0.12

*Based on 2,617 individuals with at least one hospitalisation in the pre index period and 2,596 individuals in the post index period

†The regression coefficient indicates the increase or decrease in SAR associated with a one-step increase in severity of dementia. See Statistical analyses section for the linear model relating these variables.

For peer review only

Table S4: **Standardised admissions ratios (95% CI) by ICD chapters (repeat admissions excluded)***

	Chapter	All	Mild	Moderate	Severe	Coefficient†	p-value †
**A**	Infectious and parasitic diseases	1.6 (1.2-2.0)	1.0 (0.6-1.7)	1.8 (1.3-2.5)	2.2 (1.0-4.2)	0.58	0.11
**B**	Infectious and parasitic diseases	1.7 (0.9-3.1)	0.8 (0.1-3.0)	2.2 (0.9-4.5)	2.9 (0.4-10.6)	1.06	0.11
**C**	Cancers	0.6 (0.5-0.7)	0.7 (0.5-0.8)	0.6 (0.4-0.7)	0.3 (0.1-0.7)	-0.17	0.19
**D**	Benign neoplasms or diseases of the blood	1.0 (0.8-1.2)	1.1 (0.9-1.5)	1.0 (0.8-1.3)	0.5 (0.2-1.1)	-0.32	0.22
**E**	Endocrine, nutritional and metabolic diseases	2.0 (1.7-2.4)	2.0 (1.5-2.7)	1.9 (1.5-2.5)	2.5 (1.4-4.1)	0.27	0.39
**F**	Mental and behavioural disorders	5.8 (4.9-6.7)	5.0 (3.8-6.4)	6.2 (5.0-7.5)	6.6 (4.1-9.9)	0.8	0.19
**G**	Diseases of the nervous system	2.5 (2.1-2.9)	2.3 (1.7-2.9)	2.6 (2.1-3.3)	3.1 (1.9-4.7)	0.4	0.03
**H**	Diseases of the eye	0.6 (0.6-0.8)	0.6 (0.5-0.8)	0.7 (0.6-0.9)	0.5 (0.2-0.8)	-0.07	0.65
**I**	Diseases of the circulatory system	1.2 (1.1-1.2)	1.3 (1.1-1.5)	1.2 (1.1-1.4)	1.1 (0.8-1.4)	-0.11	0.08
**J**	Diseases of the respiratory system	1.7 (1.5-1.7)	1.3 (1.1-1.6)	1.8 (1.6-2.0)	2.2 (1.8-2.8)	0.45	0.03
**K**	Diseases of the digestive system	1.0 (0.9-1.0)	1.1 (1.0-1.3)	0.9 (0.8-1.1)	0.7 (0.4-1.0)	-0.25	0.06
**L**	Diseases of the skin	1.1 (0.9-1.4)	1.1 (0.8-1.5)	1.0 (0.7-1.4)	1.8 (1.0-2.9)	0.34	0.42
**M**	Diseases of the musculoskeletal system	0.9 (0.8-1.1)	1.0 (0.8-1.3)	0.9 (0.7-1.1)	1.0 (0.7-1.5)	-0.01	0.95
**N**	Diseases of the genitourinary system	1.8 (1.7-1.8)	1.6 (1.4-1.9)	1.9 (1.7-2.1)	2.2 (1.7-2.8)	0.28	0.01
**R**	Symptoms and sign not elsewhere classified	1.9 (1.7-1.9)	1.9 (1.7-2.1)	1.9 (1.7-1.9)	1.7 (1.3-2.1)	-0.12	0.29
**S**	Injury, poisoning and certain other external causes	2.3 (2.1-2.3)	2.3 (2.0-2.7)	2.2 (1.9-2.4)	2.4 (1.8-3.1)	0.03	0.86
**T**	Injury, poisoning and certain other external causes	1.5 (1.3-1.9)	1.5 (1.1-2.1)	1.8 (1.3-2.3)	0.7 (0.2-1.7)	-0.37	0.49
**Z**	External causes	1.1 (0.9-1.3)	1.5 (1.2-1.9)	0.9 (0.7-1.2)	0.8 (0.4-1.4)	-0.34	0.22
Total		1.4 (1.4-1.4)	1.4 (1.3-1.4)	1.4 (1.4-1.4)	1.4 (1.3-1.4)	0.01	0.49

*Based on 2,596 individuals in the post index period †The regression coefficient indicates the increase or decrease in SAR associated with a one-step increase in severity of dementia. See Statistical analyses section for the linear model relating these variables.

For peer review only

For peer review only - http://bmjopen.bmj.com/site/about/guidelines.xhtml

STROBE Statement—Checklist of items that should be included in reports of ***cohort studies***

	Item No	Recommendation	Page number
**Title and abstract**	1	(*a*) Indicate the study's design with a commonly used term in the title or the abstract	2
		(*b*) Provide in the abstract an informative and balanced summary of what was done and what was found	2
**Introduction**			
Background/rationale	2	Explain the scientific background and rationale for the investigation being reported	5
Objectives	3	State specific objectives, including any prespecified hypotheses	5
**Methods**			
Study design	4	Present key elements of study design early in the paper	6
Setting	5	Describe the setting, locations, and relevant dates, including periods of recruitment, exposure, follow-up, and data collection	6
Participants	6	(*a*) Give the eligibility criteria, and the sources and methods of selection of participants. Describe methods of follow-up	6
		(*b*) For matched studies, give matching criteria and number of exposed and unexposed	Not relevant
Variables	7	Clearly define all outcomes, exposures, predictors, potential confounders, and effect modifiers. Give diagnostic criteria, if applicable	6-8
Data sources/ measurement	8*	For each variable of interest, give sources of data and details of methods of assessment (measurement). Describe comparability of assessment methods if there is more than one group	*6-8*
Bias	9	Describe any efforts to address potential sources of bias	Not relevant
Study size	10	Explain how the study size was arrived at	Not relevant
Quantitative variables	11	Explain how quantitative variables were handled in the analyses. If applicable, describe which groupings were chosen and why	6-8
Statistical methods	12	(*a*) Describe all statistical methods, including those used to control for confounding	9-10
		(*b*) Describe any methods used to examine subgroups and interactions	10
		(*c*) Explain how missing data were addressed	Not relevant
		(*d*) If applicable, explain how loss to follow-up was addressed	8
		(*e*) Describe any sensitivity analyses	10
**Results**			
Participants	13*	(a) Report numbers of individuals at each stage of study—eg numbers potentially eligible, examined for eligibility, confirmed eligible, included in the study, completing follow-up, and analysed	9
		(b) Give reasons for non-participation at each stage	9
		(c) Consider use of a flow diagram	9

For peer review only

For peer review only - http://bmjopen.bmj.com/site/about/guidelines.xhtml

Descriptive data	14*	(a) Give characteristics of study participants (eg demographic, clinical, social) and information on exposures and potential confounders	11
		(b) Indicate number of participants with missing data for each variable of interest	Not relevant
		(c) Summarise follow-up time (eg, average and total amount)	11
Outcome data	15*	Report numbers of outcome events or summary measures over time	11
Main results	16	(*a*) Give unadjusted estimates and, if applicable, confounder-adjusted estimates and their precision (eg, 95% confidence interval). Make clear which confounders were adjusted for and why they were included	12-20
		(*b*) Report category boundaries when continuous variables were categorized	
		(*c*) If relevant, consider translating estimates of relative risk into absolute risk for a meaningful time period	
Other analyses	17	Report other analyses done—eg analyses of subgroups and interactions, and sensitivity analyses	12-20 and supplementary
**Discussion**			
Key results	18	Summarise key results with reference to study objectives	21
Limitations	19	Discuss limitations of the study, taking into account sources of potential bias or imprecision. Discuss both direction and magnitude of any potential bias	23
Interpretation	20	Give a cautious overall interpretation of results considering objectives, limitations, multiplicity of analyses, results from similar studies, and other relevant evidence	24
Generalisability	21	Discuss the generalisability (external validity) of the study results	4, 23
**Other information**			
Funding	22	Give the source of funding and the role of the funders for the present study and, if applicable, for the original study on which the present article is based	25

*Give information separately for exposed and unexposed groups.

Note: An Explanation and Elaboration article discusses each checklist item and gives methodological background and published examples of transparent reporting. The STROBE checklist is best used in conjunction with this article (freely available on the Web sites of PLoS Medicine at http://www.plosmedicine.org/, Annals of Internal Medicine at http://www.annals.org/, and Epidemiology at http://www.epidem.com/). Information on the STROBE Initiative is available at http://www.strobe-statement.org.

For peer review only
